# Exosome Processing and Characterization Approaches for Research and Technology Development

**DOI:** 10.1002/advs.202103222

**Published:** 2022-03-25

**Authors:** James J. Lai, Zoe L. Chau, Sheng‐You Chen, John J. Hill, Katalin V. Korpany, Nai‐Wen Liang, Li‐Han Lin, Yi‐Hsuan Lin, Joanne K. Liu, Yu‐Chung Liu, Ruby Lunde, Wei‐Ting Shen

**Affiliations:** ^1^ Department of Bioengineering University of Washington Seattle WA 98195 USA; ^2^ Department of Mechanical Engineering University of Washington Seattle WA 98195 USA; ^3^ Department of Materials Science and Engineering National Tsing Hua University Hsinchu 30013 Taiwan; ^4^ Department of Mechanical Engineering National Taiwan University Taipei City 10617 Taiwan; ^5^ Department of Engineering and System Science National Tsing Hua University Hsinchu 30013 Taiwan; ^6^ Department of Biomedical Engineering and Environmental Sciences National Tsing Hua University Hsinchu 30013 Taiwan

**Keywords:** analytical characterizations, exosomes, exosome clinical trials, extracellular vesicles, Good Manufacturing Practices, isolation processes, MISEV2018 guidelines

## Abstract

Exosomes are extracellular vesicles that share components of their parent cells and are attractive in biotechnology and biomedical research as potential disease biomarkers as well as therapeutic agents. Crucial to realizing this potential is the ability to manufacture high‐quality exosomes; however, unlike biologics such as proteins, exosomes lack standardized Good Manufacturing Practices for their processing and characterization. Furthermore, there is a lack of well‐characterized reference exosome materials to aid in selection of methods for exosome isolation, purification, and analysis. This review informs exosome research and technology development by comparing exosome processing and characterization methods and recommending exosome workflows. This review also provides a detailed introduction to exosomes, including their physical and chemical properties, roles in normal biological processes and in disease progression, and summarizes some of the on‐going clinical trials.

## Graphical abstract

The capability to manufacture high quality exosomes is essential for fully realizing their potential as disease biomarkers as well as therapeutic agents. This review informs exosome research and technology development by comparing exosome processing and characterization methods and recommending exosome workflows. The figure shows relative yield, purity, and throughput of different exosome isolation techniques.

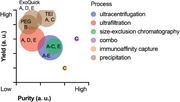

## 1 Introduction

Exosomes are extracellular vesicles (EVs) produced in endosomes of eukaryotic cells and have attracted attention in life sciences research and biotechnology because they participate in intercellular communication in various normal and pathological functions. For example, exosomes circulating in blood carries cargos such as microRNA^[^ ^1^ ^]^ that can be used in the diagnosis, prognosis, and even treatment of cardiovascular diseases (CVDs).^[^ ^2^ ^]^ Sensing technologies have been utilized to profile central nervous system derived exosomes, which are capable of crossing the blood‐brain barrier^[^ ^3^ ^]^ and carrying biomolecules between cells,^[^ ^4^ ^]^ for the early diagnostics of neurodegenerative diseases and also as a route to study the progression mechanism.^[^ ^4^ ^]^ In human immunodeficiency virus (HIV), exosomes also present utility as carriers of potential biomarkers and demonstrate potential drug delivery pathways.^[^ ^5^ ^]^ To appreciate the exosome potential and the associated challenges, this review covers biology/biochemistry, clinical applications, isolation approaches, and characterizations. This review utilizes cardiovascular diseases, neurodegenerative diseases, and infectious diseases as the foundation to discuss the disease associated exosomal pathways, and to provide a landscape of the existing exosome related clinical trials and in‐depth trial summary for demonstrating the potential of exosome technologies for real clinical applications. To realize exosomes’ potential utility as biomarkers and therapeutic agents in various biomedical fields, the ability to manufacture and characterize exosomes is crucial to realizing this potential.^[^ ^6^ ^]^ However, unlike other biologics, such as proteins, exosomes lack quality‐based methods for routine processing, and analytical characterization. Additionally, the field is lacking standard, well‐characterized reference exosome materials, which would be essential for exosome research, technology development, and manufacturing. Therefore, the review provides a brief overview of isolation techniques, introducing processing parameters and optimized methods, and conducts a comparison study of each different sample type, based on yield and purity, guiding readers to choose suitable isolation approaches. A workflow, including sample types, isolation approaches, and characterizations, was developed by following the minimal information for studies of extracellular vesicles (MISEV) guideline. The characterization section gives a comprehensive overview of analytical techniques, which are mapped to different exosome properties, and summarizes some emerging technologies. The review also discusses isolation and characterization approaches for studying or diagnosing cardiovascular diseases, neurodegenerative diseases, and infectious diseases. The discussion of exosome therapeutics and diagnostics in tandem with an in‐depth exploration of exosome isolation and characterization provides a well‐rounded focus for individuals seeking to gain a better understanding or start within the field of exosome study and application.

## 2 What is an Exosome?

Exosomes are EVs produced in the endosomes of eukaryotic cells.^[^ ^7^ ^]^ Due to the heterogeneity between and within exosome types and the overlap in characteristics between exosomes and other EVs, it is difficult to define exosomes in a way that distinguishes them from other EVs.^[^ ^8^ ^]^ This section explores the defining features of exosomes—their biogenesis and biochemical properties and how these properties differ from those of others EVs such as microvesicles and apoptotic bodies. This section also introduces the functions of exosomes in normal biological processes via their surface proteins and as carriers of molecular cargo such as nucleic acids from parent to target cells.

### 2.1 Biogenesis of Exosomes and Other EVs

**Scheme 1:**
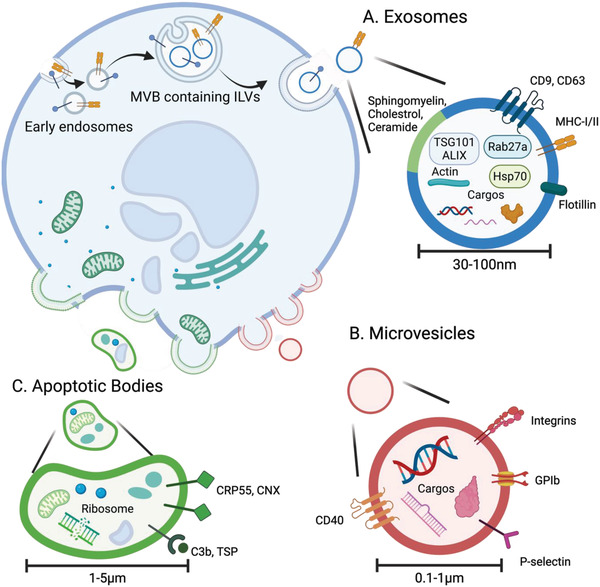
The biogenesis pathways and biochemical composition of A) exosomes, B) microvesicles, and C) apoptotic bodies. (A) Proteins, lipids, and genetic material are loaded into ILVs which are eventually released from the parent cell as exosomes. (B) Microvesicles are formed by directly budding off of the plasma membrane and contain proteins, lipids, and genetic materials. (C) Apoptotic bodies bud directly from the plasma membrane during apoptosis, and consequently, contain higher amounts of disintegrated organelle content. Created with BioRender.com.

EV subtypes are differentiated by their biogenesis. The primary pathway of exosome secretion involves fusion of exosome‐containing endosomes with the plasma membrane;^[^ ^7^ ^]^ by contrast, the secretion of microvesicles and apoptotic bodies occurs via direct budding from the plasma membrane.^[^ ^9^ ^]^ Exosome biogenesis begins with inward budding of the plasma membrane to form early endosomes (**Scheme** **1**A),^[^ ^10^ ^]^ followed by maturation into multivesicular bodies (MVBs), which involves formation of intraluminal vesicles (ILVs) via inward budding of the endosomal membrane. ILVs contain lipids, proteins, and nucleic acids from their parent cells.^[^ ^11^ ^]^ ILV formation is driven by the protein complex endosomal sorting complex required for transport (ESCRT) and associated proteins ALG2‐interacting protein X (ALIX) and tumor susceptibility gene 101 (TSG101), which group ubiquitylated MVB membrane proteins and induce inward budding of the MVB membrane.^[^ ^11^ ^]^ An alternate ILV formation pathway is the formation of lipid raft microdomains by ceramide lipids, which induces MVB membrane budding.^[^ ^12^ ^]^ Exosome secretion can be either constitutive (via the trans‐Golgi network) or regulated (via MVB fusion with the plasma membrane).^[^ ^10^ ^]^ Docking of MVBs to the plasma membrane is mediated by Rab proteins (Rab27a/b), and the fusion of endosomal and plasma membranes is mediated by SNARE proteins.^[^ ^13^ ^]^ By contrast, microvesicle release is simpler than exosome secretion and involves budding from the plasma membrane without exocytosis (Scheme 1B).^[^ ^9b^ ^]^ Microvesicle budding and pinching off is thought to involve ESCRT‐dependent viral‐like budding, or budding due to a cytoskeletal imbalance arising from lipid translocation.^[^ ^14^ ^]^ Apoptotic bodies bud directly from the plasma membrane during late apoptosis following disintegration of cell content (Scheme 1C),^[^ ^9a^ ^]^ and have high cellular and organelle content, a distinguishing feature that is useful for their isolation.^[^ ^15^ ^]^

### 2.2 Biochemical Composition of Exosomes and Other EVs

EVs are also distinguished by their physicochemical properties, allowing the detection and isolation of specific EV subtypes based on differences in properties.^[^ ^16^ ^]^ The databases Vesiclepedia (http://microvesicles.org/) and Exocarta (http://www.exocarta.org) are collections of published data on EV properties that facilitate EV isolation and analysis.^[^ ^17^ ^]^ Here we discuss the biochemical compositions of different EV subtypes and how these properties can be used to distinguish exosomes from other EVs such as microvesicles and apoptotic bodies.

#### 2.2.1 Proteins

Individual EVs typically contain less than 100 total proteins.^[^ ^18^ ^]^ Roughly 1 µg of total protein can be obtained from ≈10^9^–10^10^ EVs.^[^ ^19^ ^]^ Protein cargos are heterogeneous even between EVs of the same type,^[^ ^20^ ^]^ but some proteins are consistently found in specific EV types due to the roles of these proteins in EV biogenesis and protein sorting.^[^ ^21^ ^]^ Thus, exosomes, microvesicles, and apoptotic bodies can be distinguished based on their protein content, and proteins are used as markers in clinical diagnostics and EV characterization.^[^ ^22^ ^]^ In exosomes, characteristic proteins include CD9, CD63, and CD81, which are tetraspanin proteins commonly found on exosome membranes, and play roles in membrane fusion, signaling, and protein trafficking.^[^ ^23^ ^]^ ALIX, flotillin, and TSG101 are also involved in exosome biogenesis.^[^ ^24^ ^]^ ESCRT and the associated proteins hepatocyte growth factor‐regulated tyrosine kinase substrate (Hrs), flotillin, TSG101, and ALIX mediate MVB formation and ILV engulfment and are common exosome cargo. Rab27a and annexin are vesicle fusion and transport proteins that mediate docking of MVBs to the plasma membrane and modulate exosome secretion.^[^ ^13^, ^25^ ^]^ The cytoskeletal proteins actin and myosin and the heat shock proteins Hsp70 and Hsp90 are also enriched in exosomes.^[^ ^26^ ^]^ Collectively, these proteins serve as markers that distinguish exosomes from other EVs and can be used in exosome detection and isolation.

Other EVs possess their own characteristic protein cargo which can be used to further distinguish exosomes. Microvesicles contain proteins that generally have a higher level of post‐translational modification.^[^ ^27^ ^]^ Microvesicle membrane proteins include CD40, integrins, glycoprotein Ib, and P‐selectin.^[^ ^28^ ^]^ Tumor microvesicle membranes contain the small GTP‐binding protein ARF6, which mediates protein selection in tumor cells.^[^ ^29^ ^]^ Apoptotic bodies contain both cytoplasmic and nuclear proteins, and thus possess more complex protein cargo compositions than other EVs.^[^ ^30^ ^]^ Apoptotic body membranes contain calreticulin (CRP55) and calnexin (CNX), which promote efferocytosis and the formation of phagolysosomes, as well as receptors for thrombospondin (TSP) and the complement protein C3b, which bind TSP and C3b and promote the recognition of apoptotic bodies by phagocytes.^[^ ^31^ ^]^ These proteins serve as markers to further distinguish exosomes from microvesicles and apoptotic bodies.

#### 2.2.2 DNA and RNA

EVs carry nucleic acids to target cells where they are transcribed and translated and influence cell behavior.^[^ ^24^, ^32^ ^]^ For example, mitochondrial DNA transferred by exosomes into breast cancer cells may contribute to hormonal therapy‐resistant breast cancer by restoring the metabolic activity of impaired breast cancer cells following hormonal treatment.^[^ ^33^ ^]^ mRNA and microRNA (miRNA) are commonly transported by EVs in normal and pathological cell–cell communication.^[^ ^34^ ^]^ DNA and RNA carried by EVs reflect the parent cell conditions—exosomes from virus‐infected cells contain viral RNA, and exosomes from malignant cancer cells possess miRNA profiles that are distinct from those of normal cells.^[^ ^35^ ^]^

Due to the heterogeneity of nucleic acid composition within EV subtypes,^[^ ^36^ ^]^ establishing a genetic profile for each EV subtype is difficult. Still, there are a few consistent differences in nucleic acid composition among EV subtypes. mRNA and miRNA are enriched in microvesicles and exosomes,^[^ ^37^ ^]^ whereas ribosomal RNA (rRNA) is enriched in apoptotic bodies.^[^ ^38^ ^]^ DNA in microvesicles and exosomes often contains a complete genome,^[^ ^39^ ^]^ whereas DNA in apoptotic bodies is fragmented.^[^ ^40^ ^]^ Exosomes exhibit a variety of miRNA profiles with up to ≈500 different miRNA molecules per exosome.^[^ ^41^ ^]^ The genetic profiles and nucleic acid content of exosomes and how these differ from other EVs remain poorly defined,^[^ ^42^ ^]^ and there is a need for a large‐scale comparison of EVs to establish differences in nucleic acid composition that can be used to improve and standardize the isolation of exosomes from other EVs.

#### 2.2.3 Lipids

The lipid content of EVs is less well characterized than their protein and nucleic acid content. Hundreds of lipid species in EV membranes have been identified by mass spectrometry.^[^ ^43^ ^]^ In platelet‐derived exosomes, the most common lipids are cholesterol (42.5% of lipids), phosphatidylcholine (15.9%), and sphingomyelin (12.5%), and their derivatives.^[^ ^44^ ^]^ Interestingly, lipid compositions differ widely between exosomes and their parent cells,^[^ ^44^, ^45^ ^]^ but to a lesser extent between exosomes from a single cell line.^[^ ^44a^ ^]^ The higher content of sphingomyelin, disaturated lipids, and cholesterol in the plasma membrane of exosomes than in their parent cells may contribute to the rigidity of exosomes and their resistance to degradation, which enable them to act as effective carriers of proteins and nucleic acids.^[^ ^27b^ ^]^ The distribution of lipids between the inner and outer leaflets of the exosome membrane is unequal, which contributes to exosome stability.^[^ ^46^ ^]^ Unlike exosomes, microvesicles have a lipid content that is similar to that of their parent cells, and apoptotic bodies have a higher phosphatidylserine content than their parent cells.^[^ ^47^ ^]^

Lipids also have a regulatory function in EVs.^[^ ^37^ ^]^ A well‐known example is that phosphatidylserine in the outer membrane of apoptotic bodies acts as an “eat me” signal for phagocytes.^[^ ^30^, ^48^ ^]^ Additionally, lipids can impact inflammation: ceramide phosphates in exosomes in bronchoalveolar lavage fluid membranes play an anti‐inflammatory role following exposure to second‐hand smoke.^[^ ^49^ ^]^ Lipids are indispensable to EV function, and an extensive analysis of lipids in EVs may help identify additional physicochemical properties with which to isolate exosomes from other EV subtypes.

### 2.3 Exosome Functions

EVs perform a variety of functions in biological pathways. For example, microvesicles participate in cell signaling, apoptotic bodies transfer the contents of apoptotic cells to healthier cells,^[^ ^50^ ^]^ and exosomes participate in intercellular communication in various normal and pathological functions.^[^ ^51^ ^]^ Here, we describe examples of the function of exosomes in their natural state. Section 2 will discuss exosome in disease pathways, and clinical applications of exosomes in diagnostics and therapeutics.

#### 2.3.1 Exosome Surface Proteins

Exosomes play key roles in the activation and suppression of the immune system,^[^ ^52^ ^]^ and can modulate immune responses via antigen presentation on their surfaces. For example, exosome membranes fuse with MHC–antigen complexes and induce antigen‐specific T cell responses in the initiation and progression of inflammation.^[^ ^53^ ^]^ The exosome surface receptors CD86 and lymphocyte function‐associated antigen 1 (LFA‐1) signal inflammatory pathways that activate immune cells.^[^ ^54^ ^]^ Exosome surface proteins also facilitate immune suppression: exosomes derived from cancer cells that express programmed death‐ligand 1 (PD‐L1), an inhibitory checkpoint molecule, can suppress the function of cytotoxic T cells^[^ ^55^ ^]^ and promote the immune escape of cancer cells. Aside from surface proteins, exosomes were also proven to carry protein, DNA and RNA cargos that can induce immune responses^[^ ^51a^ ^]^ as well as other physiological functions.

#### 2.3.2 Delivery of Cargo from Parent to Target Cells

Exosomal delivery of proteins, nucleic acids, and lipids from parent to target cells represents a newly understood route of intercellular communication.^[^ ^23^, ^56^ ^]^ Transport of exosomal cargo—especially nucleic acids—can regulate the behavior of the recipient cell.^[^ ^32^, ^57^ ^]^ For example, the miRNA cargo of mesenchymal stem cell (MSC)‐derived exosomes enables these exosomes to help repair injured myocardium, serving as an alternative to MSCs.^[^ ^58^ ^]^ MSC‐derived exosomes have even shown superior ability to prevent hypertrophy or damage than MSCs themselves in vitro, due to differences in miRNA composition between the exosomes and parental cells.^[^ ^59^, ^60^ ^]^ Exosomal delivery of soluble cytokines, growth factors, and hormones is an important mechanism of intercellular communication,^[^ ^61^ ^]^ allowing intercellular communication over large distances, for example to modulate systemic immune reactions.^[^ ^61^, ^62^ ^]^ The lipid cargo of exosomes also appears to have diverse functions such as regulating the metabolism and immune function of recipient cells.^[^ ^63^ ^]^

In the progression of cancer, the initiation of metastasis is enabled by the epithelial‐mesenchymal transition—the transformation of adhesive epithelial cells to migratory, invasive mesenchymal cells. Exosomes derived from mesenchymal cells carry distinct cargo from that of epithelial cells, and these mesenchymal‐derived exosomes can promote metastasis.^[^ ^64^ ^]^ Exosomes in the tumor microenvironment can also promote tumor growth by delivering proteins, lipids, and nucleic acids that cause immune suppression.^[^ ^65^ ^]^ For example, fatty acid cargo from tumor‐derived exosomes can suppress the immune responses of recipient dendritic cells.^[^ ^63a^ ^]^ The role of exosomes in disease pathways enabled their use in clinical applications, and specific examples will be given in Section 2.

## 3 Clinical Applications of Exosomes

Exosomal cargo delivery plays a role in disease progression. Understanding the roles of exosomes in disease pathways is essential for developing exosome‐based therapeutics and diagnostics. This section describes the roles of exosomes in cancer, cardiovascular disease, neurodegenerative disease, and HIV/acquired immunodeficiency syndrome (AIDS), followed by descriptions of clinical trials applying exosomes as diagnostics and therapeutics.

### 3.1 Roles of Exosomes in Disease Pathways

#### 3.1.1 Cardiovascular Disease

**Scheme 2:**
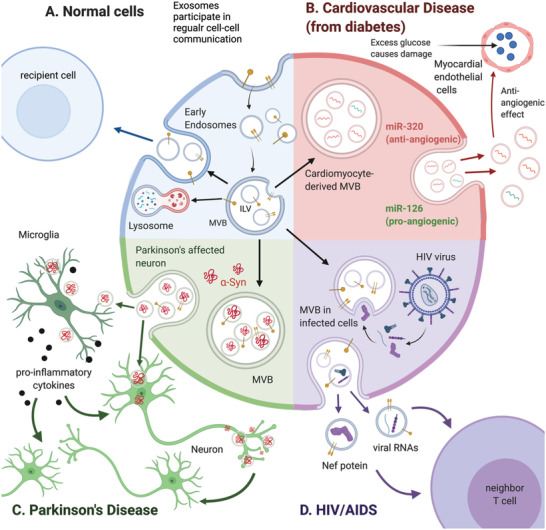
Exosome biogenesis. A) Normal biogenesis pathway. B) Exosomes derived from cardiomyocytes may play a role in the development of cardiovascular disease from diabetes by upregulating antiangiogenic miR‐320 and downregulating proangiogenic miR‐126 in neighboring endothelial cells. C) In Parkinson's disease pathogenesis, exosomes from infected neurons and microglia act as carriers to transmit *α*‐synuclein (*α*‐syn) and proinflammatory cytokines. D) HIV‐infected cells secrete exosomes containing viral genomes, antigens, and other HIV‐associated proteins. The transfer of these viral products to other cells facilitates HIV infection. Created with BioRender.com.

Exosomes contribute to the progression of cardiovascular disease in individuals with diabetes and obesity via antiangiogenic and proinflammatory effects due to their miRNA and protein content.^[^ ^66^ ^]^ Cardiomyocytes from diabetic rats secrete antiangiogenic exosomes rich in antiangiogenic miRNA 320 (miR‐320) and poor in proangiogenic miR‐126 (**Scheme** **2**B).^[^ ^67^ ^]^ This exosomal antiangiogenic activity inhibited blood vessel repair following damage due to high glucose levels, increasing the risk of cardiovascular complications. Macrophage‐derived exosomes from hypertensive rats contain low levels of anti‐inflammatory miR‐17;^[^ ^1c^ ^]^ in hypertensive humans, such macrophage‐derived exosomes may promote inflammation of human coronary artery endothelial cells, contributing to cardiovascular disease.

#### 3.1.2 Neurodegenerative Diseases

In Parkinson's disease, exosome transmission of infectious proteins induces serial cell‐to‐cell infections.^[^ ^68^ ^]^ Parkinson's disease is characterized by the histopathological formation of Lewy bodies, which consist of misfolded *α*‐synuclein.^[^ ^69^ ^]^ Exosomes may act as *α*‐synuclein carriers that host *α*‐synuclein aggregation and enable neuron‐to‐neuron, neuron‐to‐neuroglia, and neuroglia‐to‐neuroglia propagation of *α*‐synuclein (Scheme 2C).^[^ ^70^ ^]^ Exosomes containing *α*‐synuclein are taken up by neuroglia (microglial cells and astrocytes), and the *α*‐synuclein causes a neuroinflammatory response that is central in the pathogenesis of Parkinson's disease.^[^ ^70d^ ^]^ Infected neuroglia trigger neurodegeneration by secreting proinflammatory factors and additional exosomes. Thus, exosome transport of *α*‐synuclein may contribute to a malignant inflammatory cycle in the pathogenesis of Parkinson's disease.

In Alzheimer's disease, some types of exosomes appear to promote disease progression while others appear to prevent the progression.^[^ ^71^ ^]^ Exosomes are enriched in Alzheimer's disease plaques—aggregates of *β*‐amyloid protein in the brain which cause synaptic signal blocking—as evidenced by high concentrations of the exosomal protein marker ALIX in these plaques. However, exosomes derived from human cerebrospinal fluid also prevent synaptic disruption by *β*‐amyloid protein.^[^ ^72^ ^]^

#### 3.1.3 HIV/AIDS

AIDS results from infection by the HIV and its attack on the immune system.^[^ ^73^ ^]^ HIV‐infected cells are loaded with viral products, affecting the composition of exosomes derived from the infected cells (Scheme 2D).^[^ ^74^ ^]^ HIV‐related factors in exosomes appear to impact disease progression. Exosomes from HIV‐infected cells deliver negative regulatory factor (Nef) proteins to nearby CD4^+^ T cells to induce their apoptosis and depletion, a hallmark of AIDS pathogenesis.^[^ ^75^ ^]^ HIV miRNAs delivered by exosomes inhibit apoptosis, facilitate chronic inflammation, and enhance viral transcription,^[^ ^76^ ^]^ all of which promote disease progression.

### 3.2 Exosomes in Clinical Trials

**Figure 1:**
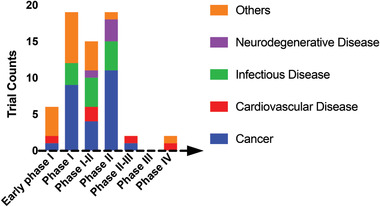
Clinical trials involving exosomes, based on an analysis of clinical trials listed in clinicaltrial.gov (May 25th, 2021; search bar: “Other terms”; keyword: “Exosome”; exclusion: trials without FDA‐defined phases). More than 40% of the trials are cancer‐related (phase I–III); other trials (color coded) are investigating exosomes as diagnostic and therapeutic agents for mild cognitive impairment, Alzheimer's disease, heart failure, stroke, periodontitis, and other conditions.

As the roles of exosomes in disease pathways have become clear, exosomes have been increasingly developed for disease treatment and diagnosis. Although there is no FDA‐approved clinical exosome product, the number of ongoing clinical trials involving exosome‐based therapeutics and diagnostics is increasing. **Figure** **1** summarizes 63 clinical trials applying exosomes to diseases listed in ClinicalTrials.gov as of June 6, 2021 (search bar: “Other terms”; keyword: “Exosome”; exclusion: trials without FDA‐defined phases). A large fraction (26/63) of the trials are cancer‐related; others are investigating exosomes as diagnostic and therapeutic agents for a variety of diseases including cognitive impairment, Alzheimer's disease, heart failure, stroke, and periodontitis, illustrating the breadth of exosome clinical applications. Eight clinical trials apply exosomes to COVID‐19 and are in phases I and II only 16 months after the onset of the pandemic, showing that exosome technology can rapidly address emerging clinical needs.

This section discusses ongoing clinical trials involving exosomes as therapeutic agents, biomarkers, and targets of therapy. Applications of exosomes to cancer therapy and diagnosis have been discussed extensively elsewhere;^[^ ^77^ ^]^ briefly, exosomes have been used for therapeutic uses to target cancer stem cells, activate immune response, enhancing antitumor immunity, and for diagnosing or monitoring patient responses following therapy for gastrointestinal, lung and breast cancers.^[^ ^78^ ^]^ Here we focus on the application of exosomes to other diseases and the challenges involved in these applications, for example, due to the complexity of bodily fluids and sample‐to‐sample heterogeneity, and the limitations of current exosome assays.

### 3.3 Therapeutics

Immune cell‐derived exosomes can promote immune responses to fight diseases,^[^ ^79^ ^]^ and stem cell‐derived exosomes can protect injured tissues during regeneration.^[^ ^80^ ^]^ The clinical trial NCT01159288^[^ ^81^ ^]^ used dendritic cell‐derived exosomes which contain antigen presentation MHC class I/II and costimulatory molecules^[^ ^82^ ^]^ as immunotherapy agents for nonsmall cell lung cancer patients following chemotherapy. In this trial, exosome treatment promoted the antitumor activity of natural killer cells.^[^ ^81b^ ^]^ Other studies have also shown that exosomes produced by disease‐ or infection‐related cells can promote immune responses.^[^ ^83^, ^84^ ^]^ The cargo composition of exosomes derived from lung carcinoma‐derived A549 cells was altered after infection with respiratory syncytial virus, and treatment with these exosomes enhanced cytokine and chemokine release and promoted an innate immune response.^[^ ^84^ ^]^ These examples demonstrate the utility of exosomes in novel therapies against diseases and injuries. Exosome‐based treatment strategies can be classified as direct, indirect, or alternative approaches, and are described in detail below.

#### 3.3.1 Direct Approach: Exosomes as Therapeutic Agents

Direct therapeutic approaches use exosomes as drug carriers to treat diseases or injuries, exploiting exosomes’ ability to transfer proteins and nucleic acids between cells. In the clinical trial NCT03384433,^[^ ^85^ ^]^ subjects with acute ischemic stroke were treated with exosomes derived from MSCs. The microRNA miR‐124, which ameliorates brain injuries by promoting neurogenesis,^[^ ^86^ ^]^ was transfected into MSC‐derived exosomes and these exosomes improved neural functional recovery and promoted neurogenesis and angiogenesis following stroke.^[^ ^87^ ^]^ Physiological adverse events including brain edema, seizures, and stroke recurrence were monitored in a 12‐month follow‐up period. The exosomes provided better stability, immune tolerability, efficacy via systemic delivery, and dosing than traditional cell therapy.^[^ ^88^ ^]^

Exosomes can serve as drug carriers that can access the central nervous system by passing through the blood‐brain barrier due to their small size.^[^ ^89^ ^]^ For instance, experiments have shown that neurogenesis promotion and cognitive function recovery could be observed in AD mice treated with MSCs‐generated exosomes, and the therapeutic use of exosomes derived from human umbilical cord MSCs was approved to mitigate neuroinflammation via the microglia activation, which leads to a better therapeutic efficacy on AD‐suffering mice via the repairment of cognitive dysfunctions and the further elimination of amyloid beta protein (A*β*) deposition.^[^ ^90^ ^]^ Based on these trial results, biotechnology companies are developing MSC‐derived exosomes for the treatment of Alzheimer's disease (Celltex Therapeutics),^[^ ^91^ ^]^ neural stem cell‐derived exosomes for penetrating the blood‐brain barrier (Aruna Bio),^[^ ^92^ ^]^ and an exosome‐based vaccine platform for preventing infectious diseases (Codiak BioSciences).^[^ ^93^ ^]^

#### 3.3.2 Indirect Approach: Exosomes as Biomarkers

In the context of biogenesis research, it was discovered that the dynamic nature and molecular profile of exosomes could be used as indicators of diseases and other clinical conditions and as predictors of treatment efficacy.^[^ ^94^ ^]^ Exosomes are found in the circulation and in all body fluids, and exhibit high stability during isolation over a large range of temperatures.^[^ ^95^ ^]^ These properties reduce the cost of storage and transportation, increasing the clinical value of exosomes as therapeutics and biomarkers. Researchers have collected and analyzed exosomes from various biopsies to investigate disease pathogenesis, to evaluate the efficacy of clinical treatments, and to seek better therapeutic strategies. For example, the clinical trial NCT03034265^[^ ^96^ ^]^ involved isolating and analyzing urinary exosomes from 24 hypertension patients to identify difficult‐to‐treat hypertension, a condition distinguished by uncontrolled blood pressure even after administration of two or more antihypertensive drugs.^[^ ^97^ ^]^ This condition is caused by abnormal renal sodium concentration,^[^ ^98^ ^]^ which is regulated by the renin–angiotensin–aldosterone system; therefore, this system was evaluated by measuring hormone angiotensin (Ang) peptides such as Ang II in patient plasma.^[^ ^99^ ^]^ Urinary exosomes were listed as a primary outcome measure since they act as indicators of renal function and thus provide criteria for measuring drug therapeutic effects. The rationale is that exosomes are secreted by tubular epithelial cells into the urine,^[^ ^100^ ^]^ and contain sodium channels such as the Na–Cl cotransporter, epithelial sodium channel subunits, and Na–K–Cl cotransporter type 2, which are targets of the hypertension drugs furosemide and thiazide. Thus, exosome properties allow evaluation of renal performance and drug sensitivity.^[^ ^100^ ^]^ This clinical study demonstrates a promising application of exosomes as biomarkers in the clinical diagnosis of a disease. To date, several researches verified the importance of exosomes as either the therapeutic agent for the treatment or the biological indicator after the therapy against diseases, particularly even other cardiovascular conditions.^[^ ^101^ ^]^ For example, doxorubicin‐induced cardiac injuries and cardiomyocyte pyroptosis could be greatly alleviated with the use of embryonic stem cell‐produced exosomes by blocking caspase‐1‐dependent cell death and elevating levels of M2 macrophages as well as anti‐inflammatory cytokines;^[^ ^102^ ^]^ on the other hand, the enhanced secretion of antihypoxic cardiac progenitor cell‐derived exosomes could be a reliable biomarker as well as the reasonable mechanism for the treatment of ticagrelor, an oral selective and reversible non‐thienopyridine P2Y_12_ inhibitor.^[^ ^103^ ^]^ These studies demonstrate the versatility of exosomes to serve, in tandem, as biomarkers and also as therapeutics.

#### 3.3.3 Alternative Approaches: Eliminating Disease Promoting Exosomes

Another therapeutic strategy is eliminating exosomes that promote diseases.^[^ ^104^ ^]^ This strategy aims to prevent disease progression by terminating the circulation of exosomes that contain detrimental disease‐related cargo such as viral miRNA and proteins,^[^ ^105^ ^]^ immunosuppressive factors that promote tumor metastasis,^[^ ^106^ ^]^ or tumor growth signals that counteract therapeutic agents.^[^ ^104^ ^]^ The clinical trial NCT04453046^[^ ^107^ ^]^ involved participants with head and neck squamous cell carcinoma (HNSCC) that were treated with a blood filtration device (Hemopurifier) on Days 1 and 21 to remove immunosuppressive exosomes from circulation. This treatment had previously been shown to remove viral particles from the plasma of hepatitis C and HIV patients,^[^ ^108^ ^]^ and had demonstrated therapeutic efficacy in a clinical trial (NCT04595903), which aimed to remove SARS‐CoV‐2 virus from the circulation of COVID‐19 patients.^[^ ^109^ ^]^ In the HNSCC study, subjects were also treated with pembrolizumab, an FDA‐approval monoclonal antibody for first‐line treatment of HNSCC.^[^ ^110^ ^]^ During this combinatorial treatment, circulating exosomes were targeted for removal, and were also used as an indicator to assess the efficacy of the treatment. The kinetics of exosome concentration depletion and recovery before, during, and after treatment were monitored as secondary outcome measures and to evaluate therapeutic efficacy.

Other studies have removed detrimental disease‐related exosomes by suppressing exosome biogenesis using drugs as inhibitors.^[^ ^104^, ^111^ ^]^ For example, the chemotherapeutics tipifarnib and ketoconazole elicited a dose‐dependent decrease in exosome biogenesis and secretion in C4‐2B cells and PC‐3 cells.^[^ ^112^ ^]^ These exosome‐inhibiting drugs are being tested in an ongoing clinical trial for treating patients with HNSCC (NCT03719690),^[^ ^113^ ^]^ and in another trial for preoperative treatment of patients with recurrent glioma or breast cancer brain metastases (NCT03796273).^[^ ^114^ ^]^ These studies indicate that removing detrimental exosomes by using external devices such as the Hemopurifier or by using exosome biogenesis inhibitors may be a feasible therapeutic strategy.

### 3.4 Diagnostics

Exosomes have great potential utility for clinical diagnosis.^[^ ^95^, ^115^ ^]^ Current clinical trials use exosomes as diagnostic biomarkers based on their roles in intercellular communication and disease progression, in addition to their related cargo and surface proteins.^[^ ^116^ ^]^ For example, as described above (Section 2.1.2), exosomes from infected macroglia carry *α*‐synuclein and proinflammatory cytokines and contribute to the progression of Parkinson's disease.^[^ ^70d^ ^]^ Exosomes also play a significant role within cancer diagnostics as their surface and internalized biomarkers (e.g., miRNA, proteins) act as indicators for the disease, a topic discussed substantially elsewhere.^[^ ^95^ ^]^ Thus, this section details clinical trials that utilize exosomes as diagnostics for other conditions including cardiovascular disease, neurodegenerative diseases, and immune reconstitution inflammatory syndrome (IRIS). These examples were chosen based on the disease prevalence in exosome‐based clinical trials, and on the novelty of the diagnostic approaches used in each case. This section concludes with recommended validation characteristics for diagnostic exosome‐based assays.

#### 3.4.1 Cardiovascular Disease

The ongoing clinical study NCT03478410 is using exosome quantity and cargo to characterize atrial fibrillation due to high blood pressure, atherosclerosis, and heart abnormalities.^[^ ^117^ ^]^{Verdecchia, 2018 #980} This study is investigating how exosomal mRNA and miRNA alter myocardial cell gene expression. To elucidate the relationship between cardiovascular‐derived exosomes and atrial fibrillation, the trial is examining whether exosomes released from the epicardial fat of patients with and without atrial fibrillation vary, and is evaluating exosomes as biomarkers for arrhythmia, for prevention and treatment.

#### 3.4.2 Neurodegenerative Diseases

No diagnostic assay currently exists for Parkinson's disease, and definitive diagnoses can be made only after death and autopsy.^[^ ^118^ ^]^ Exosomes show promise for use as biomarkers for early diagnosis of Parkinson's disease and other neurodegenerative disorders such as Alzheimer's disease.^[^ ^4^ ^]^ Exosomes participate in the progression of Parkinson's disease via transport of *α*‐synuclein as described above (Section 2.1).^[^ ^70^, ^119^ ^]^ Exosome content and leucine‐rich repeat kinase 2 (LRRK2) are potential biomarkers that being explored in Parkinson's disease research; mutations in LRRK2 are a cause of the disease.^[^ ^119^ ^]^ The clinical trial NCT01860118 (completed but results not yet posted) compared exosomal proteins and blood and urine biomarkers from Parkinson's disease patients and healthy participants (*n* = 601) to establish an assay that evaluates the effects of LRRK2 inhibitors.^[^ ^120^ ^]^ This trial is attempting to develop the first diagnostic assay for Parkinson's disease by using exosome biomarkers.

Current assays for diagnosing Alzheimer's disease involve positron emission tomography scans, cerebrospinal fluid (CSF) protein content evaluation, and detection of beta‐amyloid protein aggregates with mass spectrometry. Alternate methods to diagnose Alzheimer's disease are needed. Exosomes contribute to the progression of Alzheimer's disease and may serve as therapeutic targets. The ongoing clinical trials NCT03275363^[^ ^121^ ^]^ and NCT03944603^[^ ^122^ ^]^ are longitudinal studies examining the behavior of exosomes in patients at risk for Alzheimer's disease. NCT03944603 has a primary outcome measure of changes in blood and cerebrospinal fluid exosomal markers every two years in individuals aged 60–89. By examining the relationship between aging and immune system biomarkers, this study aims to provide insight into mechanisms underlying cognitive decline and development of Alzheimer's disease.

#### 3.4.3 Immune Reconstitution Inflammatory Syndrome in TB‐HIV Coinfected Patients

Exosomes are potentially useful in immune disease diagnostics due to their miRNA cargo.^[^ ^123^ ^]^ Host cell miRNAs target HIV genes and can be used to characterize the HIV disease phenotype.^[^ ^124^ ^]^ However, the role of miRNAs in acute infection and co‐infection with both HIV and TB remains unclear. IRIS is a paradoxical state in which a patient's condition worsens due to repaired immunity, and is exemplified by HIV patients who undergo antiretroviral therapy (ART).^[^ ^125^ ^]^ In patients coinfected with TB and HIV, IRIS is a particular concern as the treatment of one condition may worsen the other.^[^ ^126^ ^]^ Currently there is no IRIS assay and diagnosis of IRIS is complex.^[^ ^125^ ^]^

To develop an IRIS assay and to understand the role of exosome miRNA transport in IRIS, the ongoing clinical trial NCT03941210 profiles miRNA expression in HIV/TB‐coinfected IRIS patients to examine exosomes as a potential predictive and prognostic biomarker for IRIS.^[^ ^127^ ^]^ Of 134 participants, 74 were TB‐HIV‐coinfected (37 TB‐IRIS, 37 non‐TB‐IRIS), 20 were HIV‐ART‐naïve, 20 had newly active TB, and 20 were healthy. Plasma and exosome miRNA samples were profiled by flow cytometry. To address the time‐consuming and specialized nature of miRNA detection, this study used flow cytometry for high‐throughput screening of exosomes.

#### 3.4.4 Validation of Exosome‐Based Diagnostic Assays

Assays that examine exosomal cargo and exosomal biomarkers can be used in disease detection.^[^ ^115^ ^]^ FDA guidelines regulate the evaluation of diagnostic assays before clinical use in the U.S.,^[^ ^128^ ^]^ to ensure that the quality of exosome‐based diagnostic assays matches that of approved diagnostic assays. Recommended validation characteristics required for exosome‐based diagnostic assays are listed below, based on the Clinical and Laboratory Standards Institute (CLSI) guidelines EP09c, EP15‐A3, EP05‐A3, EP7‐A3, EP17‐A2, EP28‐A3C, and EP6‐A.^[^ ^129^ ^]^

Validation characteristics for exosome‐based diagnostic assays are as follows.

- Accuracy: How closely an assay's analyte readout compares to the sample's true analyte concentration. Difficulties in accuracy arise when an exosome subtype is not clearly defined and can be addressed by using recovery studies in which a known exosome quantity is added to a controlled sample.
- Precision: How closely the results of multiple independent experiments agree. Precision is concentration‐dependent, and the use of multiple concentrations of target exosome biomarkers is advised.
- Analytical sensitivity: The smallest amount of substance in a sample that can accurately be measured by an assay. Determined by the limit of blank, limit of detection, and limit of quantification.
- Analytical specificity: Measures whether an assay captures the correct biomarker. It is important to be aware of the presence of vesicles that are similar to the target exosome, or the presence of inhibitory compounds in the assay.
- Reference interval: The range of values usually found in individuals without the assayed disease or condition.
- Linearity and range: Linearity is used to establish an assay's range—the span of test results that can be accurately determined—and is determined by using different dilutions of target exosome (analyte).
- Sample type: Exosomes are found in diverse sample types including blood, urine, and cerebral spinal fluid. Sample quality can vary significantly within a given sample type depending on analyte purity and concentration.

## 4 Challenges, Guidelines, and Workflow for Exosome Processing and Characterizations

### 4.1 Challenges

The development of new exosome‐based diagnostic assays and therapeutics is challenging due the limitations of current methods for isolating and characterizing exosomes.^[^ ^130^ ^]^ Developing robust methods to isolate exosomes is difficult because of the overlap or similarity of physicochemical properties between EV subtypes. **Table** **1** lists the most common measurable physicochemical properties of EVs including diameter, morphology, density, and protein markers, and includes centrifugation rates used to isolate these EV subtypes. The typical size ranges of exosomes (30–100 nm diameter) and microvesicles (100–1000 nm) are very similar and often overlap, thus, there is not a clear distinction that can be made based on vesicle size.^[^ ^131^ ^]^ In addition, the density ranges of the different EVs overlap significantly. The morphology of EVs after isolation is affected by the methods used in sample preparation and characterization.^[^ ^132^ ^]^ Exosomes derived from different cell types or from the same cell type under different conditions can display different surface markers; for example, CD63 is an accepted exosome marker but is missing in certain exosome subpopulations.^[^ ^133^ ^]^ For these reasons, isolating exosomes is challenging when using techniques such as ultracentrifugation, ultrafiltration, size‐exclusion chromatography, precipitation, and immunoaffinity capture. Therefore, it is important to precisely define and describe EV subtypes, and to regulate characterization and isolation methods. As suggested in the 2018 update of the MISEV2018,^[^ ^130^ ^]^ researchers are urged to use operational terms for EV subtypes that refer to physical characteristics with defined ranges, biochemical composition (e.g., CD63^+^/CD81^+^), and to explicitly reference the parent cell or location of origin.

**Table 1:** Identifying properties of EVs

	Exosomes	Microvesicles	Apoptotic bodies
Centrifugation speed [g]	100 000–200 000	10 000–20 000	2000
Density [g mL^−1^]	1.10–1.21	N/A	1.16–1.28
Diameter [nm]	30–100	100–1000	1000–5000
Morphology	Round/cup‐shaped, homogeneous	Heterogeneous	Heterogeneous
Protein markers	ESCRTs, CD9, CD63, TSG101, ALIX, MHC‐I/II, Hsp70	CD40, CD62, selectins, integrins, flotillin‐2, KIF23, CEE1L/CAS, RACGAP1	Annexin V, TSP, C3b, histones, CNX, CRP55

### 4.2 MISEV2018 Guidelines

Obtaining high‐quality exosomes is essential for developing diagnostics and therapeutics, but most isolation and analytical characterization techniques have not been developed specifically for exosomes. Therefore, the International Society for Extracellular Vesicles updated MISEV to guide exosome preparation and characterization.^[^ ^130^ ^]^ MISEV2018 categorizes information as “required,” “should be provided if possible,” or “alternative if all recommendations cannot be followed.” MISEV2018 includes EV collection and preprocessing, separation and concentration, characterization, functional studies, and reporting requirements. Functional studies are application‐specific and vary widely and will not be included here. Preprocessing, isolation, and characterization are discussed in Sections 4 and 5. MISEV2018 encourages submitting detailed experimental protocols for isolation and characterization to EV‐TRACK (evtrack.org), which provides an aggregate measure of the level of detail for the proposed experiments from which authors can gauge whether additional steps are needed for their experiments.

### 4.3 High Quality Standards and Scale‐Up Production

Exosome research and technology development also needs high‐quality reference specimens for use as standards to establish new analytical approaches. These reference specimens must have known purity and well‐characterized surface markers and cargo, and should be as close to 100% pure and homogeneous as possible. However, this is a chicken‐and‐egg situation because robust isolation processes and analytical characterization methods are needed to produce pure exosome references, and vice versa. Currently, a reference specimen can be generated by collecting exosomes from cell culture media; however, batch‐to‐batch variation is unknown, and the heterogeneous nature of exosomes is a concern. Issues with commercially available exosome specimens include mismatch of exosome types and incomprehensive analytical characterization.

Versatile isolation approaches to facilitate exosome production is also a challenge. For example, ultrafiltration is a core method for isolating high‐quality exosomes in bulk due to its ease of use, scalability, and ability to isolate exosomes with high purity and defined size. Ultrafiltration is limited by membrane clogging and vesicle trapping; this issue can be addressed by tangential flow filtration, but this approach is limited by low processing volume. These issues must be resolved to make ultrafiltration a method of choice for exosome isolation. Size‐exclusion chromatography allows high‐quality exosome preparation and excellent reproducibility and has great potential for high‐throughput industrial applications, especially because the gravity flow used in size‐exclusion chromatography (SEC) causes minimal damage to exosome structure and function.^[^ ^134^ ^]^

### 4.4 Workflow for Exosome Processing and Characterization

**Scheme 3:**
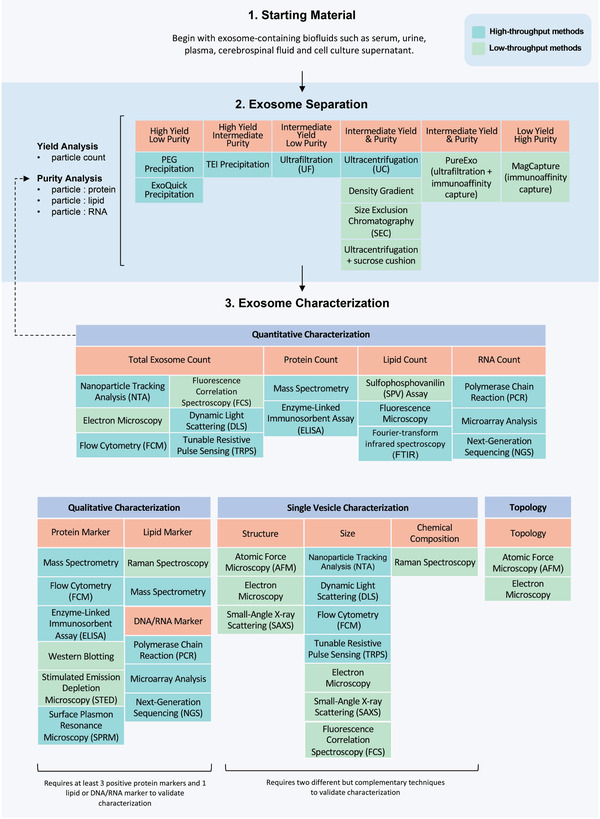
Exosome processing and characterization flowchart. The exosome separation portion of the workflow includes common processing approaches with yields and purity levels. The characterization portion of the flowchart contains quantitative characterization, qualitative characterization, single vesicle characterization, and topology. The workflow starts with measuring and recording parameters of the starting materials such as cell count, fluid volume, and quantity of non‐EV molecules. Next, an appropriate processing method is selected based on the starting material and the target yield and purity. After exosome isolation, characterization begins with quantitation of the purity and yield of particles, proteins, lipids, and nucleic acids. If the sample does not meet the desired purity and yield, additional processing is required. Appropriate methods are then selected for bulk and single vesicle characterization to finish the characterization process.

Obstacles to clinical applications of exosomes also include the lack of unified workflows and standards of characterization. To address this limitation, we created a workflow for exosome processing and characterization (**Scheme** **3**) by distilling the recommendations of MISEV2018^[^ ^130^ ^]^ and based on an analysis of current exosome processing and characterization techniques (described in Sections 4 and 5, respectively). The exosome separation portion of the workflow includes common processing approaches with high yields and purity levels (Section 4). The characterization portion of the flowchart contains the four types of EV characterization listed in MISEV2018: 1) quantitative characterization, 2) qualitative characterization, 3) single vesicle characterization, and 4) topology. The workflow starts with measuring the parameters of the starting materials, such as cell count, fluid volume, and quantity of non‐EV molecules. Next, an appropriate processing method is selected based on the starting material and the target yield and purity. After exosome isolation, characterization begins with quantitation of purity and yield, and of proteins, lipids, and nucleic acids. If the sample does not meet the desired purity and yield, additional processing is required. Appropriate methods are then selected for bulk and single vesicle characterization to finish the characterization process.

## 5 Exosome Processing and Isolation

Important parameters to consider in exosome processing and isolation are the composition of the starting sample, the basis of the separation method, and how these factors affect the quality and characteristics of the products. Ideally, processing will generate exosomes of high purity and yield that are useful for enabling life science research as well as diagnostic and therapeutic applications. Isolation techniques are compared in depth in Section 4.3.

### 5.1 Sample Types

The nature of the starting material has a large impact on the exosome processing methods selected and their efficacy.^[^ ^135^ ^]^ Starting materials for exosome isolation include cell culture media, plasma, serum, urine, saliva, CSF, and milk.^[^ ^136^ ^]^ Different starting materials require different processing approaches. Plasma is a complex biological fluid that contains exosomes along with cell debris, apoptotic bodies, microvesicles, and plasma proteins, all of which complicate exosome isolation due to their overlapping size and biochemical properties.^[^ ^137^ ^]^ Urine has fewer interfering particles than blood or plasma, but a lower exosome concentration^[^ ^138^ ^]^ and therefore requires larger volumes for an equivalent yield. Cell culture media obtained following the culture of cells is widely utilized for exosome mass production because it is simple, inexpensive, and does not require animal or human subjects.^[^ ^139^ ^]^ Cell culture media can have a higher exosome yield than serum or plasma; CSF has the lowest exosome content.^[^ ^140^, ^141^ ^]^ A thorough comparison study of exosome isolation across different sample types would provide a useful reference for exosome research and technology development.

### 5.2 Exosome Isolation Techniques

**Table** **2** summarizes five common exosome processing techniques: ultracentrifugation, ultrafiltration, precipitation, immunoaffinity capture, and size‐exclusion chromatography. These methods vary in the resulting exosome purity and yield and are often used in combination. For each technique, we discuss the physicochemical basis of exosome isolation, protocols, and commercial applications. Microfluidic devices are not included in this table but are discussed in Section 4.2.6.

**Table 2:** Exosome processing techniques

Process	Time [min]	Sample types	Volume [mL]	Advantages	Disadvantages	Ref.
Ultracentrifugation	140–600	CCM, urine	≤25	Good purity for clinical treatment applications and proteomic studies	Impurities (e.g., protein aggregates) Expensive instrument Complex procedure Repetitive steps damage isolated vesicles	[137, 142, 148, 164]
Ultrafiltration	130	CCM, urine	≤15	High throughput Wide range of sample volume Simple procedure	Low purity Reduced yield by filter clogging Not suitable for plasma	[137, 142, 164, 172]
Precipitation	30–120 or overnight	CCM, plasma	≤10	High yield High throughput Simple procedure	Low purity (e.g., polymer contamination) Not suitable for plasma	[137, 142, 148, 164, 172]
Immunoaffinity capture	240	CCM	0.5–3	High purity for proteomic analyses High selectivity	Low yield Low sample volume Expensive Extra elution step	[148]
Size‐exclusion chromatography	15	CCM	0.5–1.5	Higher purity than precipitation Low required volume Versatile for various specimen types Preserves vesicle integrity	Protein contamination Low yield Expensive instruments and column Complex procedure Dilution is required for viscous samples	[137, 142, 148, 172]

#### 5.2.1 Ultracentrifugation

Ultracentrifugation is the gold standard for exosome isolation and is used in 80% of exosome processing.^[^ ^142^ ^]^ Ultracentrifugation does not require elaborate sample preparation and is inexpensive except for the initial instrument cost. However, it is time‐consuming and achieves only moderate exosome purity. Ultracentrifugation separates sample components based on density;^[^ ^143^ ^]^ however, there is substantial overlap in the density ranges of different EV types. Large centrifugal forces separate samples into layers; high density particles settle to the bottom of a tube, while lower density particles move to the top. Ultracentrifugation speeds used in exosome isolation range from 100 000 × *g* to 210 000 × *g*.^[^ ^144^ ^]^ Increasing the speed can improve separations but risks damaging the exosomes.^[^ ^145^ ^]^

Differential centrifugation is an ultracentrifugation technique that involves multiple rounds of centrifugation to separate target exosomes from cell debris, larger vesicles, and proteins.^[^ ^146^ ^]^ The procedure demands frequent user intervention to remove supernatants and pellets and to set up spin cycles. Exosomes can be lost during the repeated removal of supernatant and transfers of sample between tubes; therefore, loss of exosome is expected and larger sample volumes are used at the start of processing to obtain a desired yield.^[^ ^147^ ^]^ Despite these shortcomings, differential centrifugation is well‐tested and consistently produces exosomes of moderate yield and purity.^[^ ^148^ ^]^

Using media with a density gradient in conjunction with ultracentrifugation can improve exosome isolation.^[^ ^134^ ^]^ Density‐gradient ultracentrifugation, also called isopycnic ultracentrifugation, uses a set of preconstructed discontinuous density layers to facilitate exosome isolation between two distinct sucrose or iodixanol layers.^[^ ^70c^ ^]^ Zonal ultracentrifugation is a type of density‐gradient ultracentrifugation that uses a gradient of lower densities to separate particles based on size.^[^ ^146^ ^]^ In contrast with differential centrifugation, zonal ultracentrifugation usually requires only one extended (up to 18 h) high‐spin cycle.^[^ ^149^ ^]^ Density‐gradient ultracentrifugation results in higher purity exosome products than differential centrifugation^[^ ^150^ ^]^ but has lower yield and throughput. It is also labor‐intensive and expensive due to the construction of the polymer density layers. For these reasons, density‐gradient ultracentrifugation is typically not recommended for large‐scale exosome processing.

#### 5.2.2 Ultrafiltration

Ultrafiltration is defined by the use of extremely small pores (≈100 nm diameter) and can be used to isolate exosomes based on size.^[^ ^151^ ^]^ Ultrafiltration methods are rapid—one round of filtration lasts from seconds to 30 min—allowing high throughput. Ultrafiltration isolates vesicles by applying pressure to drive sample fluid through membranes with ≈100 nm pores.^[^ ^152^ ^]^ Membranes with smaller or larger pore sizes can be used in additional steps to remove other unwanted particles. The method is faster than ultracentrifugation, but the applied pressure can damage exosomes due to shear stress, and can result in loss of exosomes due to membrane adhesion and membrane blockage from accumulation of particles,^[^ ^152^ ^]^ reducing exosome yield and prolonging processing time.^[^ ^153^ ^]^ Strategies such as membrane washing^[^ ^154^ ^]^ have been developed to mitigate these issues.

Exosome ultrafiltration methods include sequential filtration, tandem filtration, centrifugal ultrafiltration, and tangential flow filtration.^[^ ^152^, ^155^ ^]^ Sequential and tandem filtration are dead‐end filtration techniques performed with a syringe. Sequential filtration is defined by multiple rounds of filtration each with a different molecular weight cutoff; tandem filtration combines multiple filters in a single syringe. The size‐exclusion limits for exosomes are typically 20–200 nm; in tandem filtration, exosomes are captured in a middle membrane.

Centrifugal ultrafiltration combines dead‐end filtration and centrifugation to separate exosomes through nanoscale pores. A nanoporous membrane fixed inside a tube is spun, applying a centrifugal force that pushes sample content through the membrane.^[^ ^156^ ^]^ Centrifugal ultrafiltration is typically preceded by preliminary centrifugation or dead‐end filtration at 0.22 µm to remove large particles such as cells, cell debris, and protein aggregates, and to prevent clogging.

Tangential flow filtration (TFF) was recently adopted for exosome isolation.^[^ ^155^ ^]^ Unlike the above approaches, TFF does not apply pressure orthogonal to the membrane, but instead passes samples tangentially to the membrane. This approach avoids membrane blockage due to buildup of particles on the membrane. TFF can be used to process larger volumes of fluid with higher reproducibility than ultracentrifugation techniques,^[^ ^155^ ^]^ and is gentler on the sample. However, the processing time is longer for TFF than for other filtration methods.

#### 5.2.3 Precipitation

Precipitation methods are widely used in EV characterization. A worldwide survey showed that precipitation is the preferred process for EV RNA analysis.^[^ ^157^ ^]^ Precipitation methods use volume‐excluding polymers to tie up water molecules and force less soluble components out of solution.^[^ ^158^ ^]^ Biological materials are excluded from the solvent regions occupied by these polymers and are concentrated until their solubility is exceeded, at which point precipitation occurs. The approach is commonly used in conjunction with other isolation methods. Although this method results in higher yield, the lower product purity is a limitation.^[^ ^159^ ^]^

Since the introduction of polyethylene glycol (PEG) in 1964,^[^ ^160^ ^]^ nonionic volume‐excluding polymers such as dextrans and other hydrophilic polymers^[^ ^161^ ^]^ have become the dominant precipitation agents due to their low tendency to denature proteins at high concentrations and elevated temperatures, unlike ethanol and other organic precipitating agents.^[^ ^162^ ^]^ Volume‐excluding polymer‐based precipitation reagents have been used in commercial exosome processing kits that do not require expensive equipment or technical expertise, such as ExoQuick (System Biosciences) and Total Exosome Isolation (Thermo Fisher).^[^ ^163^ ^]^ These kits do not achieve the highest product purity, but their benefits include flexibility, low cost, and low requirements for equipment and training. Subsequent centrifugation, filtration, or gel filtration^[^ ^164^ ^]^ of samples processed with these kits can be used to improve the purity of the exosome product.

#### 5.2.4 Immunoaffinity Capture

Exosomes can be isolated based on recognition of their unique surface markers by antibodies immobilized on surfaces such as magnetic beads, chromatography column resins, multiwell plates, and microfluidic devices.^[^ ^134^ ^]^ This approach, called immunoaffinity capture, can achieve a higher specificity of exosome isolation than approaches that use physical properties. Immunoaffinity capture is often used in conjunction with preprocessing methods such as SEC^[^ ^165^ ^]^ or centrifugation^[^ ^166^ ^]^ to remove protein aggregates and other large particles. Sample types such as plasma, whole blood, and cell‐culture media contain particles that can reduce the specificity of exosome capture by occluding antigen binding sites or promoting nonspecific binding if left in large quantities.

Immunoaffinity capture is a gentle process that retains the biological activity of the exosomes after isolation.^[^ ^134^ ^]^ A nonantibody‐based process was developed with TIM‐4 and Ca+ dependent binding, which could capture and elute out exosomes without exposing the sample to nonphysiological environments.^[^ ^134^ ^]^ In general, the technique is limited by antibody availability and exhibits low yield due to the small sample volumes that can be processed.^[^ ^134^ ^]^ In addition, a long incubation period is required. For example, the commonly used magnetic Dynabeads (Thermo Fisher) require two 12 h incubation periods, one for conjugation of antibodies and the other for bead capture.^[^ ^165^ ^]^ These lengthy incubation periods are required due to the large bead size (1.0–4.5 µm),^[^ ^167^ ^]^ their low mobility in solution, and their low surface‐area‐to‐volume ratio. One approach to speed up this process is to use temperature‐ or pH‐responsive magnetic nanoparticles,^[^ ^168^ ^]^ which reduces the incubation and separation times to only a few minutes due to the much larger surface‐area‐to‐volume ratio of the nanoparticles (40× larger for 25 nm nanoparticles than for 1 µm Dynabeads) and greater magnetophoretic mobility, following aggregation induced by changes in temperature or pH, to allow rapid magnetic separation.

Raman scattering based immunoaffinity approaches also exploit magnetic properties to isolate and characterize exosomes. Raman scattering can be used to identify molecules via their specific chemical fingerprints. Magnetic surface‐enhanced Raman scattering has been used to detect breast cancer in patient samples with high sensitivity and specificity.^[^ ^169^ ^]^ The integration of characterization with sample processing is vital to streamlining therapeutic and diagnostic uses of exosomes, and distinguishes this approach from other immunoaffinity methods.

#### 5.2.5 Size‐Exclusion Chromatography

SEC is the gentlest chromatography technique and is widely used for isolation and purification of biopolymers such as proteins and polysaccharides. Isolation of exosomes by SEC preserves vesicle integrity and biological activity and has high yield.^[^ ^6d^ ^]^ SEC separates biomolecules based on differences in hydrodynamic radius as they pass through an unreactive, low‐adsorption resin consisting of a porous matrix of beads packed in a column.^[^ ^170^ ^]^ Particles larger than the pores elute first, while smaller particles and molecules penetrate the pores to varying degrees based on their size, with elution time increasing with decreasing particle or molecule size. To obtain high‐resolution particle size, processing parameters such as column dimensions, bead packing, type of resin, flow rate, and system volume are important factors to consider.^[^ ^171^ ^]^ This method can be applied to separate samples across a range of viscosities, from low viscosity urine and cell culture media to high viscosity plasma. However, pretreatment of samples by ultracentrifugation or ultrafiltration is necessary to obtain EV preparations free of protein and lipoprotein impurities.^[^ ^6e^ ^]^

To simplify EV isolation by SEC, commercial prepacked columns are available, such as qEV (Izon Science)^[^ ^172^ ^]^ and HiLoad Superdex (GE Healthcare).^[^ ^142^ ^]^ SEC with prepacked columns results in a lower exosome recovery rate and a more heterogeneous EV population than EV isolation by precipitation, but is fast, convenient, reproducible, applicable to many sample types, and does not require a chromatography system as it can be used with a standalone pump. However, SEC results in low‐concentration samples, and an additional enrichment step is often required.

#### 5.2.6 Microfluidics

Microfluidic devices can combine multiple processes such as immunoaffinity capture, filtration, application of acoustics or electrical waves, and field flow fractionation into one single‐step device with multiple channels that isolates exosomes with high automation and reproducibility.^[^ ^173^ ^]^ Precise control over fluid flow through these channels ensures laminar flow, which has more predictable fluid dynamics than turbulent flow. Microfluidic devices are compact; for example, the immunoaffinity‐based ExoChip device is 75 mm × 25 mm^[^ ^174^ ^]^ and can be scaled up easily by adding sampling wells. Its compact size makes the device portable and easily stored in tightly packed lab spaces. Microfluidics is well‐suited for diagnostic applications due to the sample sizes (nanoliters to microliters). Microfluidics devices can also include exosome detection modules to integrate exosome isolation and characterization.^[^ ^174^ ^]^

Microfluidics devices have included arrays of silicon‐nanowired micropillars that trap exosomes based on size exclusion.^[^ ^175^ ^]^ As fluid passes through the device, exosomes are trapped in the openings. This process is rapid, but the trapped exosomes must be eluted out of the pores, which can take up to 24 h, a time‐consuming step which limits the utility of this process in diagnostics. Separation by size has also been accomplished by viscoelastic‐based isolation.^[^ ^176^ ^]^ Using serum or cell culture media, exosomes can be sorted based on elastic lift forces with high sensitivity and specificity. Samples are mixed with elastically responsive biocompatible polymers and are deposited into viscoelastic media. As the fluid flows through the device, larger particles and extracellular molecules which exhibit higher elastic forces from the media are driven away from the exosomes.

Acoustic microfluidic approaches have been developed to separate exosomes based on size.^[^ ^173b^ ^]^ Acoustic waves are gentler than micropillar arrays and involve less contact. Waves are generated throughout a flowing sample by interdigital transducers. The wave frequency determines the particle size cutoff for separation at two channels. One channel contains waste such as apoptotic bodies and larger microvesicles, while the other contains only exosomes. The run time of a single sample is only 25 min, and because it is contact‐free, exosomes maintain their biological activity. In addition to acoustics, electrical waves have also been used in microfluidics to isolate exosomes in a label‐ and contact‐free manner.^[^ ^177^ ^]^

Ion‐based separation exploits the more negative charge of exosomes relative to other particles.^[^ ^178^ ^]^ A microfluidic device developed by Mogi et al. uses two inlet channels and two outlet channels with high and low voltage to separate positively and negatively charged particles. One channel from each pair is high voltage and attracts negatively charged particles, while the other channel is low voltage and attracts positively charged particles. A perpendicular ion channel in the center creates an ion depletion zone that pushes charged particles into the channels, while uncharged particles remain near the center. This device is calibrated for voltage and flow rate to optimize exosome retention, and has exhibited significantly better yield than ultracentrifugation.

### 5.3 Comparisons of Exosome Isolation Techniques

**Figure 2:**
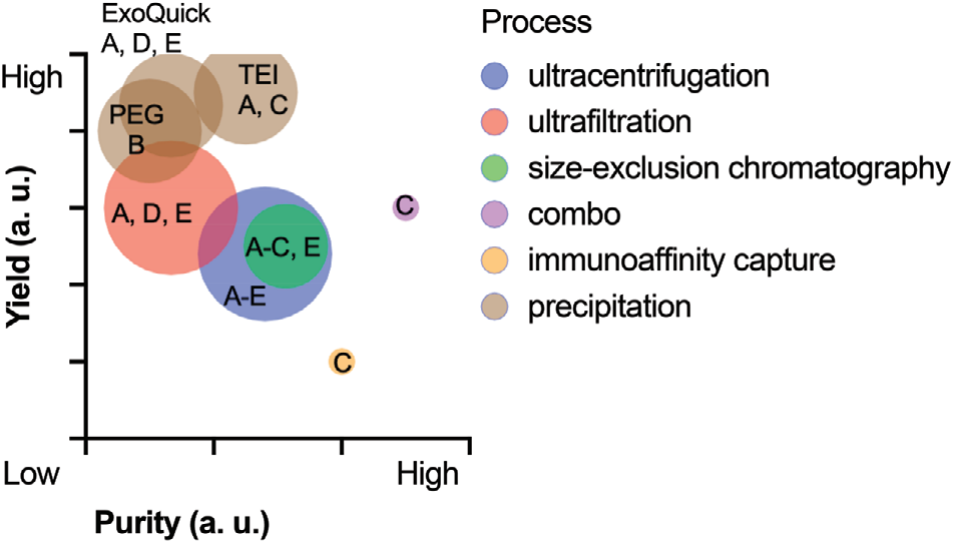
Relative yield, purity, and throughput of different exosome isolation techniques. ‐ Qualitative data were obtained from comparison studies, A–E.^[^ ^137^, ^142^, ^148^, ^164^, ^172^ ^]^ Bubble size indicates the throughput of each method relative to ultracentrifugation, the gold‐standard for exosome isolation. Precipitation methods analyzed include polyethylene glycol (PEG), Exoquick‐TC (System Biosciences), and total exosome isolation (TEI, Invitrogen).

Several studies have systemically compared common exosome isolation techniques. **Figure** **2** summarizes the results of five such comparison studies^[^ ^137^, ^142^, ^148^, ^164^, ^172^ ^]^ in terms of exosome yield and purity. Most throughput values were readily obtained from the studies, but the throughputs of ultracentrifugation and ultrafiltration must consider both processing time and sample volume (ultrafiltration has a faster processing time than ultracentrifugation but a smaller sample volume). Therefore, in Figure 2 we defined “throughput” as the volume of sample processed in a given time. Yield and purity could not be compared directly between studies, so we used relative scales, with yields ranked based on particle counts per unit volume, and with purity calculated as the ratio of total protein content in a solution to its vesicle count.^[^ ^142^, ^172^ ^]^

In the comparison study by Tian et al.^[^ ^172^ ^]^ (study A in Figure 2), exosome isolation from platelet‐free plasma was used as a model system to compare exosome yields and purities when using different commercial products. The products tested were the precipitation‐based kits ExoQuick (System Bioscience) and Total Exosome Isolation (TEI; Thermo Fisher), the SEC‐based qEVsingle kit (iZON Science), and an ultrafiltration device (Millipore). Ultracentrifugation was included for reference. The methods with the highest to lowest yields were ExoQuick, TEI, ultrafiltration, qEVsingle, and ultracentrifugation. The methods with the highest to lowest purities were ultracentrifugation, qEVsingle, TEI, ultrafiltration, and ExoQuick—nearly the reverse order as for exosome yield.

Among isolation techniques using cell culture media, precipitation had the highest yield, and a combination of precipitation and filtration (PureExo Exosome Isolation kit; Fisher Scientific) gave the highest purity. Ludwig et al.^[^ ^142^ ^]^ (study B in Figure 2) showed that precipitation gives higher yield and lower purity than other methods by comparing ultracentrifugation, precipitation with PEG, sucrose density gradient with ultracentrifugation, and size‐exclusion chromatography. Patel et al.^[^ ^148^ ^]^ (study C in Figure 2) compared exosome isolation using four commercial kits which employ different isolation techniques: precipitation (TEI, Thermo Fisher), precipitation and filtration (PureExo), immunoaffinity capture (MagCapture, Fujifilm Wako), and SEC (qEVsingle, iZON Science). The combination of precipitation and filtration by PureExo produced higher purity and yield than immunoaffinity capture, which showed higher purity but lower yield than most of the other methods.

Combinations of isolation techniques can improve exosome yield and purity, as shown above in the results for PureExo, which combines precipitation and targeted filtration. Alvarez et al.^[^ ^164^ ^]^ (study D in Figure 2) processed urinary exosomes using different methods including combinations of methods: classic ultracentrifugation, 30% sucrose density gradient ultracentrifugation, filtration with ultracentrifugation (0.22 µm filter, Millipore), ultrafiltration (Vivaspin 20, Sartorius), and precipitation (ExoQuick, System Bioscience). If urine specimens are preprocessed with dl‐dithiothreitol, the study showed that ExoQuick precipitation with a greater volume of ExoQuick‐TC reagent and higher final centrifugation speed is a suitable alternative to a larger number of samples, and purity could be improved simply by adding an extra SEC step. In another comparison study, Lobb et al.^[^ ^137^ ^]^ (study E in Figure 2) compared different isolation techniques using cell culture media and human plasma as starting materials. The results of this study indicated that repeated ultracentrifugation can damage vesicles, and that combining ultrafiltration and SEC improves purity and yield and reduces overall processing time versus repeated ultracentrifugation.

### 5.4 Exosome Isolation Techniques for Disease Applications

#### 5.4.1 Cardiovascular Diseases

Isolating exosomes from CVD patients has proven effective for diagnosis and prognosis.^[^ ^179^ ^]^ Whole blood, serum, and plasma are widely used for CVD diagnosis^[^ ^180^ ^]^ because they are easily obtained and can be used to detect CVD pathology.^[^ ^2^ ^]^ The amount of circulating EVs is positively correlated with the presence and severity of several CVDs, and exosomal proteins and miRNAs have been identified as biomarkers.^[^ ^180^ ^]^ For example, exosomal miR‐320a is upregulated in patients with chronic heart failure,^[^ ^181^ ^]^ and has an antiangiogenic effect in diabetic rats.^[^ ^67^ ^]^ Exosomes have been isolated from patient blood and plasma by using the ExoQuick kit preciptation method,^[^ ^181^ ^]^ and from culture cells and animal models by centrifugation and density gradient methods.^[^ ^1^, ^67^ ^]^ The particle count following exosome isolation can be influenced by contaminating lipoprotein particles, which exhibit similar density and size as exosomes.^[^ ^182^ ^]^ For example, low‐density lipoprotein—which transports cholesterol in blood, participates in coagulation,^[^ ^183^ ^]^ and is a risk factor for atherosclerosis and CVDs^[^ ^184^ ^]^—coprecipitates with exosomes. There is a demand for standardized methods capable of specifically isolating exosomes.

#### 5.4.2 Neurodegenerative Diseases

Exosome isolation for researching neurodegenerative diseases such as Parkinson's and Alzheimer's utilizes sample types such as brain tissue samples, plasma, and CSF.^[^ ^4^ ^]^ The sample type influences the selection of an isolation technique.^[^ ^185^ ^]^ Brain tissue homogenization results in formation of small particles, which confound determination of exosome yield and purity following exosome isolation.^[^ ^186^ ^]^ The discussion below focuses on exosome isolation from plasma and CSF for diagnosing neurodegenerative conditions.^[^ ^169^, ^187^ ^]^ In general isolation via centrifugation needs to consider specimen viscosity by diluting specimens, adjusting centrifugation speed and duration, and utilizing sucrose cushion protocols.^[^ ^185^, ^188^ ^]^

Plasma‐derived exosomes provide insight into the CNS due to their ability to cross the blood‐brain barrier into the bloodstream.^[^ ^70^, ^189^ ^]^ CNS‐specific exosomes from plasma are often isolated from non‐CNS exosomes and other biomolecules by using anti‐L1CAM antibodies,^[^ ^190^ ^]^ though one study has suggested that L1CAM is not associated with CNS‐derived exosomes in either plasma or CSF.^[^ ^191^ ^]^ Isolated CNS exosomes are further characterized by using CNS‐specific biomarkers such as tau and *α*‐synuclein.

CSF passes through the blood‐brain barrier after being in contact with brain tissue, and exosomes in CSF can reflect changes in brain tissue pathologies that are otherwise difficult to observe. Similarly, CSF comes into contact with the spinal cord and meninges and can reflect pathologies of these CNS elements. CSF is a primary sample type used for CNS disease diagnosis,^[^ ^192^ ^]^ but CSF‐specific exosome isolation methods are needed to achieve sufficient purity. For example, confounding nonexosomal proteins must be removed during exosome isolation. One such protein is nonexosomal tau, which is associated with neurodegenerative disease when accumulated in specific regions of the brain,^[^ ^193^ ^]^ and can confound the study of exosome–cell communication.^[^ ^188^ ^]^ Other such proteins include abundant CSF proteins such as albumin and immunoglobulin, which can obscure scarcer proteins that are important in exosome‐based diagnostics.^[^ ^192^ ^]^ To address these issues, a novel microfluidic device was developed^[^ ^192^ ^]^ that uses negative pressure oscillation to streamline exosome isolation, and enabled the identification of more exosome‐related proteins than traditional ultracentrifugation and PEG‐based precipitation methods. Novel methods such as this microfluidics approach that improve the isolation of CSF‐derived exosomes are critical for clinical sample analysis and neurodegenerative diagnostic research.^[^ ^194^ ^]^

#### 5.4.3 Infectious Diseases

Exosomes isolated from patients with infectious diseases such as tuberculosis and HIV/AIDS reflect the disease state in the body.^[^ ^195^, ^196^ ^]^ Exosome isolation techniques and challenges have been outlined below for HIV/AIDS. Nef proteins secreted by exosomes derived from HIV‐infected cells trigger T‐cell death and promote disease progression, making exosome isolation a valuable tool in determining HIV patient prognosis.^[^ ^197^ ^]^ Exosomes are typically isolated from patient plasma by centrifugation,^[^ ^197^, ^198^ ^]^ which results in contamination with HIV viral particles. Other isolation methods include differential ultracentrifugation from cell culture,^[^ ^199^ ^]^ precipitation of exosomes from plasma and CD4^+^ T‐cell culture,^[^ ^200^ ^]^ and density‐dependent ultracentrifugation and immunoaffinity purification of exosomes from plasma, all of which also result in contamination with HIV viral fragments.^[^ ^201^ ^]^ An acetylcholinesterase (AChe) assay can be used to select for exosomes from a centrifuged sample;^[^ ^201^ ^]^ AChe differentiates HIV fragments from human exosomes and is inexpensive.^[^ ^202^ ^]^

## 6 Exosome Characterization

Exosome characterization methods overlap with respect to the exosome properties they examine. To design a characterization process, begin with Scheme 3, which provides a flowchart summarizing exosome processing and characterization methods, then obtain more information about the specifications and advantages/disadvantages of techniques of interest from **Table** **3** (quantitative exosome analysis methods), **Table** **4** (qualitative exosome characterization methods), and **Table** **5** (single vesicle characterization methods). Characterization methods are discussed in detail below.

**Table 3:** Quantitative exosome analysis methods

	Method	Advantages	Disadvantages	Throughput	Refs.
Total exosome count	Nanoparticle tracking analysis	Minimal sample preparation Rapid Samples are reusable	High sample purity required Not suitable for polydispersed particles	High	[148, 174, 177, 203]
	Electron microscopy (Cryo‐EM)	Highly specific with immunogold labeling	Sample preparation with immunogold labeling	No	[205]
	Flow cytometry	Accurate count	Not suitable for particles ≤200 nm	High	[203d]
	Fluorescence correlation spectroscopy	Accurate count	Small sample volume (10^−15^ L) High sample concentration and purify required	High	[177, 203]
	Dynamic light scattering	Rapid (minutes) Sample are reusable	Not suitable for polydispersed particles Bias for larger particles Minimum sample concentration required	High	[133, 148, 177, 203, 279]
	Resistive pulse sensing		High sample purity required	High	[133]
Protein	Mass spectroscopy	High specificity Multiplexed protein identification	High sample purity required	High	[235, 280]
	ELISA	High sensitivity and specificity Commercially available	Limited by antibody availability	High	[206, 281]
Lipid	Sulfophosphovanilin assay	Lower cost	Minimum >50 µg mL^−1^ lipid Low sensitivity	No	[216]
	Fluorescence microscopy with lipophilic dye	Visible	Calibration with standards Prone to photobleaching	High	[217]
	Fourier‐transform infrared spectroscopy	High accuracy and reproducibility Rapid Low cost Small sample amount	Not sensitive for cholesterol and other sterols	High	[213, 214, 282]
DNA/RNA	PCR	High sensitivity and accuracy	Limited multiplex capability	High	[225]
	Microarray	Direct detection	Bias for longer sequences	High	[222, 225]
	Next generation sequencing	Multiplexed analysis Small sample input Reading short fragments	Time consuming Restrained by an intrinsic error rate	High	[225, 229]

**Table 4:** Qualitative exosome characterization methods

	Method	Advantages	Disadvantages	Throughput	Refs.
Protein	Western blot	Cost‐effective	Limited sensitivity Antibody availability	No	[225, 229, 281]
	Flow cytometry	Intact particle phenotyping Multiplexed protein marker detection	Not suitable for particles ≤200 nm	High	[203, 232]
	Stimulated emission depletion microscopy	High‐resolution imaging High specificity	Time consuming Antibody availability	No	[203, 234, 235]
	Surface plasmon resonance microscopy	Label‐free High sensitivity	Influenced by sample concentration and particle size	High	[236]
	ELISA	High sensitivity and specificity Commercially available	Antibody availability	High	[206]
	Mass spectroscopy	Comprehensive analysis	High sample purity required	High	[235]
Lipid	Raman spectroscopy	Label‐free High specificity and sensitivity	Time consuming High sample purity required	No	[236, 237]
	Mass spectroscopy	Label‐free High specificity	High sample purity required	High	[235]
DNA/RNA	Next generation sequencing	Multiplexed analysis Small sample input Reading short fragments	Time consuming Restrained by an intrinsic error rate	High	[225, 229]
	PCR	High sensitivity and accuracy	Limited multiplex capability	High	[225]

**Table 5:** Single vesicle characterization methods

	Method	Advantages	Disadvantages	Throughput	Refs.
Structure	Electron microscopy (scanning EM)	High resolution images Elemental analysis	Sample preparation (fixation and staining) Time consuming	No	[130, 242]
	Atomic force microscopy	High vertical resolution (0.1 nm)	Sample preparation (dehydration, immobilization)	No	[175, 177, 208, 241, 245]
	Small‐angle X‐ray scattering	Simple sample preparation High resolution (1 nm)	Limited to monodispersed samples Concentration must be >10^11^ vesicles mL^−1^	No	[148, 175]
Size	Nanoparticle tracking analysis	Minimal sample preparation Rapid Sample are reusable	High sample purity required Not suitable for polydispersed particles	High	[148, 174, 177, 203]
	Dynamic light scattering	Rapid (minutes) Sample are reusable	Not suitable for polydispersed particles Bias for larger particles Minimum sample concentration required	High	[148, 177, 242, 279]
	Flow cytometry	Single particle detection	Influenced by particle aggregates Not suitable for particles ≤200 nm	High	[203, 243]
	Electron microscopy	Visible	Sample preparation (staining)	No	[177]
	Small‐angle X‐ray scattering	Simple sample preparation High resolution (1 nm)	Limited to monodispersed samples Minimum >10^11^ vesicles mL^−1^	No	[148, 175]
	Fluorescence correlation spectroscopy	Single molecule detection	Small sample volume (≈10^−15^ L) Sample concentration and purification required	High	[177, 203]
	Tunable resistive pulse sensing	Suitable for polydispersed samples	Multiple membranes needed for different exosome sizes Influenced by membrane pore size/shape, vesicle surface property Membrane clogging	High	[148, 175, 239, 246, 283]
Chemical composition	Raman spectroscopy	High specificity and sensitivity	Time consuming High sample purity required	No	[177, 237]
Topology	Atomic force microscopy	High contrast on flat samples High resolution	Influenced by vesicle immobilization	No	[175, 177, 208, 243, 245, 284]
	Electron microscopy (scanning EM)	Large depth of field No postprocessing High resolution (1–20 nm)	Sample preparation (fixation, dehydration, length process)	No	[131, 242, 285]

### 6.1 Quantitative Characterization

Quantitative characterization methods (Table 3) are used to assess the success of exosome isolation and the quality of the product—the yield and purity with respect to biomolecules such as proteins, lipids, and nucleic acids.^[^ ^147^, ^148^ ^]^

#### 6.1.1 Total Exosome Count

Exosome yield is quantified by using methods that measure particle count,^[^ ^130^ ^]^ which include nanoparticle tracking analysis (NTA), flow cytometry, fluorescence correlation spectroscopy (FCS), dynamic light scattering (DLS), resistive pulse sensing (RPS), and electron microscopy (EM).^[^ ^203^ ^]^ The most commonly used techniques are NTA and FCS.

- NTA is a high‐throughput visualization technique that monitors the Brownian motion of particles in liquid suspension.^[^ ^203a^ ^]^ Particle size and concentration are determined by analyzing video of scattered light from randomly diffusing particles under laser beam illumination.
- FCS is a high‐throughput statistical technique used to characterize molecule concentration, size, and rate of diffusion. The approach uses a laser to illuminate a very small volume (1 fL) of fluorescently labeled sample.^[^ ^204^ ^]^ The fluorescence intensity of the molecules fluctuates due to Brownian motion, and the average particle concentration can be estimated based on fluorescence intensity over time.^[^ ^205^ ^]^

DLS and RPS often overestimate total particle count, making these methods less reliable for particle count determination.^[^ ^130^ ^]^ In DLS, particles larger than exosomes produces high intensity signals that mask the lower intensity signals of exosomes, resulting in inaccurate particle counts in low purity samples.^[^ ^206^ ^]^ Also, nonexosome contaminants such as protein aggregates, lipoproteins, and other particles in the 30–100 nm size range falsely register as exosomes.^[^ ^203d^ ^]^ NTA is better for analyzing polydisperse samples, but is prone to underestimating particle count due to aggregates similar in size to exosomes.^[^ ^206^ ^]^ By contrast, quantitative measurements using flow cytometry unaffected by the presence of non‐exosome particles that are not fluorescently labeled.^[^ ^207^ ^]^ The challenge for flow cytometry in exosome analysis lies in its limited ability to analyze nanoscale particles smaller than 200 nm.^[^ ^208^ ^]^ Therefore, nanobeads such as latex or silica beads, which provide different refractive index are sometimes bound to particles to increase their surface area, resulting in greater scattering intensity.^[^ ^209^ ^]^ Fluorescence signals are influenced by cell debris and cytosolic proteins; therefore, measuring particle count accurately with flow cytometry relies on high‐purity samples.^[^ ^205^ ^]^ EM is a high‐resolution (10^−10^ m) imaging technique that is used with immunogold labeling to differentiate vesicles from nonvesicle components.^[^ ^210^ ^]^ Cryo‐EM and freeze‐fracture transmission electron microscopy are commonly used for exosome characterization because they do not require sample fixation and dehydration.^[^ ^203^, ^205^ ^]^ With recent development of nanoscale flow cytometry (nFCM), sub‐micrometer‐sized vesicles can be more accurately analyzed. The nFCM setups are usually calibrated with beads of known sizes to validate the detection limit; the refractive index range of the testing beads, too, can be selected based on the refractive index of EVs.^[^ ^211^ ^]^ In recent studies, nFCM is an important evaluation method that achieves the detection of EVs below 100 nm in size; study of a laboratory‐built nFCM utilized two single‐photon counting avalanche photodiodes to simultaneously detect the side scatter and orange fluorescence in the device setup, this extends the resolution of EV profiling to as small as 40 nm.^[^ ^172^ ^]^ The nFCM widened the applicability of conventional FCM, with detection limit comparable to electron microscopy and intrinsic phenotyping ability, and it has the potential to be widely adopted not only in identification of exosomes but also nanoparticles such as bacteria, mitochondria, FNPs, viruses, as far as to Quantum dots.^[^ ^212^ ^]^

#### 6.1.2 Protein Content

Protein content (mass) is used as an indicator to quantify the purity of exosome samples. The ratio of protein mass to total exosome particle count is used to determine sample purity.^[^ ^130^ ^]^ Mass spectrometry and enzyme linked immunosorbent assay (ELISA) are used to identify and quantify specific protein markers.

- Mass spectrometry is a high‐throughput technique used to detect molecules based on the mass‐to‐charge ratio of ions. Mass spectrometry techniques used in exosome proteomics^[^ ^213^ ^]^ involve simple sample preparation to avoid exosome damage.^[^ ^214^ ^]^ Mass spectrometry combined with bioinformatics^[^ ^215^ ^]^ allows systematic characterization of exosome‐specific proteins. For example, a study of exosome‐mediated intercellular communication between vascular smooth cells and endothelial cells used liquid chromatography and tandem mass spectrometry to identify 495 proteins involved in exosome‐mediated intercellular communication, including 261 previously unidentified proteins which were subjected to ontological analysis to reveal their functions.^[^ ^216^ ^]^
- ELISA is a common immunolabeling technique for quantitating peptides and proteins via antibody recognition.^[^ ^213^ ^]^ ELISA is used for exosome profiling and diagnostics, allowing detection of protein markers and quantitation of exosome‐specific antigens and tumor antigens on exosomes.^[^ ^217^ ^]^

ELISA is cost‐effective for some protein markers, while mass spectrometry allows protein quantitation in a complex biological sample.^[^ ^218^ ^]^ However, mass spectrometry is not an easily accessible technique in clinical research and has high technical requirements, limiting its widespread use.

#### 6.1.3 Lipid Composition

The lipid composition of exosome membranes can be used to differentiate exosome subtypes. The ratio of exosomes with specific lipids to the total exosome count is a metric for evaluating the purity of targeted exosomes.^[^ ^130^ ^]^ Lipid quantitation methods include sulfophosphovanilin (SPV) assay, fluorescence microscopy, and Fourier‐transform infrared microscopy (FT‐IR).^[^ ^219^ ^]^ SPV assays quantify lipids via a colored compound produced by reacting phosphovanillin with lipid‐derived carbonium ions in the presence of sulfuric acid.^[^ ^219^, ^220^ ^]^ The assay requires sample concentrations greater than 50 µg mL^−1^ lipid for accurate results with low variability.^[^ ^220^ ^]^ Fluorescence microscopy in conjunction with a lipophilic dye for plasma membranes such as DiR and PKH26 is also used to quantify lipids in exosomes by comparing images of exosomes with reference standards.^[^ ^221^ ^]^ FT‐IR provides lipid counts at higher accuracy, reproducibility, and speed, and at lower cost and sample volume than SPV and fluorescence microscopy.^[^ ^220^, ^221^ ^]^ However, FT‐IR lacks sensitivity for cholesterol and other sterols since it is difficult to distinguish their C–C and C–H vibrational bands from those of other molecules.^[^ ^220^ ^]^

#### 6.1.4 DNA/RNA Analysis

Exosomes enable intercellular DNA and RNA transport. The ratio between the number of targeted DNA/RNA sequences and total exosome count is also used to analyze exosome purity. Common nucleic acid quantification techniques suitable for exosome DNA/RNA analysis include microarray technologies, next generation sequencing (NGS), and PCR.^[^ ^222^ ^]^

- Microarray technologies allow analysis of the expression of thousands of genes simultaneously.^[^ ^223^ ^]^ Unknown DNA or reverse‐transcribed RNA (cDNA) fragments are labeled with fluorescent markers before hybridization with known gene sequences.^[^ ^224^ ^]^ The fluorescence emitted by bound complementary sequences enables quantification of gene expression. In a microarray study of mRNA and miRNA in bovine milk exosomes, 670 miRNAs and 43 713 mRNAs were identified.^[^ ^223^ ^]^
- NGS platforms allow high‐throughput, massively parallel sequencing.^[^ ^225^ ^]^ Current NGS platforms include sequencing by hybridization, sequencing by synthesis, pyrosequencing, and ion semiconductor sequencing.^[^ ^226^ ^]^ Illumina is the most widely used NGS platform for DNA and RNA analysis, and uses bridge amplification to generate several million dense clusters of DNA strands to undergo sequencing.^[^ ^227^ ^]^ The advantages of bridge amplification are increased sequencing throughput with shorter time spent, and an accuracy of up to 99.9%.^[^ ^227^, ^228^ ^]^ NGS techniques facilitate the identification of exosomal RNA markers by profiling exosomes with only a small input sample.^[^ ^229^ ^]^

Of these methods, PCR is the most prevalent and is the gold standard because it is highly sensitive and exhibits better accuracy than NGS and microarray approaches.^[^ ^225^ ^]^ Digital droplet PCR (ddPCR) is a recently developed method that demonstrated better reproducibility and sensitivity for exosome analysis than other PCR methods, by partitioning and encapsulating DNA segments in nanoliter droplets to enable quantitation via Poisson statistics.^[^ ^230^ ^]^ With enhanced signal‐to‐noise ratio and better sensitivity and accuracy, ddPCR has been used to detect DNA and miRNA in body fluids with low exosome content.^[^ ^231^ ^]^ However, the multiplexing capability of ddPCR and PCR in general is limited. Microarray technologies are advantageous for high‐throughput and direct detection, but the reproducibility of data remains challenging across platforms. NGS is well‐suited for multiplexed analysis but is limited by a finite intrinsic error rate caused by signal uncertainty and the lack of polymerase fidelity during replication.^[^ ^225^ ^]^

### 6.2 Qualitative Characterization

Qualitative characterization methods are used to identify exosomes and to validate proteomics, lipid identification, and DNA/RNA sequence coverage,^[^ ^232^ ^]^ and are described below and in Table 4.

#### 6.2.1 Protein Content

There is no universal protein marker for confirming the presence of exosomes. Specific exosome subtypes are identified by using a combination of protein markers that distinguish them from other EV subtypes. Western blot, flow cytometry, stimulated emission depletion (STED) microscopy, and surface plasmon resonance microscopy (SPRM) are used to qualitatively characterize exosomes in terms of their protein markers.^[^ ^165^, ^213^, ^233^ ^]^

- STED microscopy is a super‐resolution technique that visually identifies exosome protein markers.^[^ ^203^, ^234^ ^]^ STED microscopy uses two lasers, one to excite the fluorophore, the other to deactivate fluorophores in specific regions of the sample. The approach overcomes the limited resolution associated with conventional microscopy because the deactivation minimizes fluorescence at the focal point, enhancing resolution.^[^ ^235^ ^]^
- SPRM is a high‐throughput technique that can detect exosomal membrane proteins and provide real‐time data on protein binding kinetics.^[^ ^236^ ^]^ During measurement, incident polarized light couples to plasmons in the metal, and a fraction of the light is reflected or absorbed depending on the angle of incidence and the properties of the sample. The reflected light is recorded by a camera and is correlated with the presence of membrane proteins.^[^ ^237^ ^]^ Standalone plasmonic sensors have also been commonly implemented in recent years to detect exosomal membrane proteins by applying the same polarized light‐metal coupling principle and measuring the angle change of the polarized light absorbed by surface plasmon.^[^ ^238^ ^]^

Mass spectrometry can rapidly identify proteins for exosome protein marker characterization, but the sensitivity relies on high sample purity.^[^ ^213^ ^]^ Flow cytometry, ELISA, Western blotting, and STED offer high specificity via antibody recognition, but antibody availability limits the detectable protein markers.^[^ ^217^ ^]^ ELISA, Western blotting, and STED are time‐consuming, whereas flow cytometry allows rapid, high‐throughput processing.^[^ ^239^ ^]^ SPRM can be used to detect protein markers in real‐time at high sensitivity without immunolabeling,^[^ ^236^ ^]^ and may also be used to simultaneously analyze fluorescently labeled proteins. One limitation of SPRM for exosome analysis is that the output is dependent on vesicle concentration, diameter, and mean antigen density.^[^ ^236^ ^]^

#### 6.2.2 Lipid Composition

Lipids serve as alternative markers to distinguish exosomes from other EV subtypes. Exosomal lipid markers are detected using fluorescently labeled lipid‐binding proteins, Raman spectroscopy, and mass spectrometry.^[^ ^203^, ^221^ ^]^ Raman spectroscopy is low‐throughput and relies on inelastic scattering of photons to determine vibrational and rotational modes of molecules.^[^ ^240^ ^]^ A high‐intensity laser is directed at the sample and the incident light scatters as it is deflected by the sample. A small amount of light scatters at wavelengths different from the wavelength of the laser source (Raman scatter), depending on the chemical structure of the analyte. The Raman spectrometer records a spectrum with intensity peaks that correspond to specific bond vibrations. By using this technique, the types of lipid present in exosomes can be distinguished as nonprotein markers.^[^ ^241^ ^]^ Mass spectrometry is also used to distinguish different types of lipids in exosomes.^[^ ^242^ ^]^ Both Raman spectroscopy and mass spectrometry are label‐free techniques with high specificity. Raman spectroscopy requires setups such as surface‐enhanced Raman spectroscopy and laser tweezers Raman spectroscopy for exosome lipid analysis.^[^ ^243^ ^]^

#### 6.2.3 DNA/RNA Analysis

The genomic content of exosomes is analyzed using microarray technologies, NGS, and qPCR,^[^ ^176^ ^]^ as described in Section 5.1.4. The determination of DNA and RNA abundance requires high‐throughput measurements due to the complex genetic information content of exosomes, and are often conducted using microarray and NGS technologies.^[^ ^244^ ^]^

### 6.3 Single Vesicle Characterization

Single vesicle characterization methods (Table 5) focus on individual exosome characteristics including size, structure, and chemical composition. These characteristics are valuable for guiding selection of isolation methods that maximize exosome purity and yield. Single vesicle characterization is essential in evaluation of exosomes as drug carriers for therapeutic applications.^[^ ^130^ ^]^

#### 6.3.1 Structure

Exosome structure includes the molecular orientation of lipids that make up the vesicle. Structural characterization provides information on how exosomes function and interact with cellular components while traveling between cells.^[^ ^130^ ^]^ Techniques used to examine exosome structure include atomic force microscopy (AFM), small‐angle X‐ray scattering (SAXS), and scanning electron microscopy (SEM).

- AFM uses a scanning probe to interact with the surface molecules of exosomes.^[^ ^245^ ^]^ To prevent vesicle deformation or rupture, tapping mode is more commonly used than contact mode.^[^ ^203b^ ^]^ Phase contrast imaging reveals differences in sample properties such as density and viscoelasticity.^[^ ^203^, ^246^ ^]^
- SAXS characterizes exosome structures by analyzing the scattering pattern caused by a monochromatic beam of X‐rays directed at the sample.^[^ ^203b^ ^]^ This technique has been used to investigate exosome lipid bilayers, vesicle size, and the presence of soluble proteins.^[^ ^203d^ ^]^ The utility of SAXS is limited because the accuracy is affected by the high vesicle heterogeneity and low scattering properties of exosomes.

AFM and SEM produce high‐resolution images of exosomes, but sample preparation for SEM may alter exosome morphology, and the electron beams can damage exosomes. SEM can be combined with energy dispersive X‐ray spectroscopy to determine the elemental composition of exosomes via the spectrum of emitted X‐rays when the electron beam strikes the sample.

#### 6.3.2 Particle Size

Exosome size can be measured by using NTA, DLS, flow cytometry, electron microscopy, SAXS, flow cytometry, and tunable resistive pulse sensing (TRPS). TRPS is a high‐throughput, electrical zone sensing technique for quantitating particle count and size.^[^ ^203b^ ^]^ The approach utilizes two containers of conductive fluid connected by an adjustable constriction for particles of different sizes to travel through.^[^ ^247^ ^]^ Electrical current is introduced and particles traveling through the aperture cause a change in electrical current. As particles of different sizes flow through the adjustable nanopore, electrical resistance changes are measured to determine particle count and size.

Higher throughput can be achieved with NTA, DLS, flow cytometry, and TRPS than with electron microscopy, SAXS, and flow cytometry. NTA and DLS measure the hydrodynamic radii of exosome particles,^[^ ^203b^ ^]^ but this measurement may not reflect the true exosome size, since hydrodynamic radius depends on solution composition and particle surface structure, and the exact relation between hydrodynamic and geometrical diameter is typically unknown.^[^ ^203d^ ^]^ Moreover, the accuracy of size distribution measurements by NTA vary depending on surface properties and exosome diffusion behavior.^[^ ^203a^ ^]^ DLS is limited to monodisperse samples as the light scattering of polydisperse samples does not yield accurate size distributions.^[^ ^248^ ^]^ This limitation may be addressed by first separating particles of different sizes by SEC, then taking measurements of monodisperse samples with DLS.^[^ ^203d^ ^]^ TRPS, because of its adjustable pore size, is suitable for measuring polydisperse samples;^[^ ^203b^ ^]^ however, imperfect nanopore shape, vesicle surface properties, and nonspecific adhesion between particles and the membrane may contribute to measurement uncertainties.^[^ ^203d^ ^]^ Imaging flow cytometry is prone to “swarming” when measuring samples with high particle concentration, causing multiple particles to register as single larger particles.^[^ ^249^ ^]^ In addition, lipid‐based particles typically have lower refractive index, resulting in lower scattering intensity when compared to reference beads, adding uncertainty to the determination of size.^[^ ^203c^ ^]^ Low scattering intensity is also an issue for size determination by SAXS, which requires highly concentrated exosome samples (>10^11^ vesicles mL^−1^) to produce a sufficiently strong signal for measurements.^[^ ^203d^ ^]^ Electron microscopy, SAXS, and flow cytometry are beneficial for studying the size of individual particles. Size measurements with electron microscopy are straightforward and superior to those using SAXS and flow cytometry, as SAXS size determination is limited to monodisperse samples, and flow cytometry measurements use small volumes and rely on high‐purity samples.^[^ ^203^, ^205^ ^]^

#### 6.3.3 Chemical Composition

Exosome chemical composition is analyzed with Raman spectroscopy (Section 5.2.2).^[^ ^203c^ ^]^

### 6.4 Topology

Topological characterization methods (Table 5) are used to study exosome function and interactions with other biomolecules. The exosome surface contains proteins that interact with cell to alter their behavior.^[^ ^233a^ ^]^ Exosome lipid membranes fuse with cell membranes, allowing delivery of exosomal cargo including proteins and nucleic acids. AFM and SEM are useful for topological characterization. AFM force spectroscopy generates force–extension curves that indicate the presence of specific biomolecules,^[^ ^203b^ ^]^ and AFM microscopy has been used for characterizing exosome morphology and substructural organization.^[^ ^250^ ^]^ Electron microscopy techniques may be used to generate 2D or 3D images of exosome topography.^[^ ^251^ ^]^ AFM offers higher contrast on flat samples than electron microscopy, and the 3D resolution is not easily affected by the environment.^[^ ^252^ ^]^ However, SEM delivers a larger depth of field, and the direct representation of samples does not require image processing. SEM measurements must be made in a vacuum to obtain high resolution images unless environmental SEM is used.^[^ ^132^ ^]^

### 6.5 Emerging Exosome Characterization Methods

Accurate characterization of exosomal properties often requires validation using orthogonal techniques, each of which have technical challenges and limitations, and the processes used to purify samples and prepare them for each analysis often influence sample characteristics. NTA, flow cytometry, and DLS give limited accuracy when processing polydisperse samples.^[^ ^203^, ^253^ ^]^ These approaches rely on exosome specimens with high purity and require repeated measurements to acquire an accurate representation of all the particles in the sample. Electron microscopy requires fixation and dehydration which influence exosome structure and topology.^[^ ^203b,c^ ^]^ TRPS and STED have limitations due to pore clogging and nonspecific membrane binding in TRPS, and photobleaching and phototoxicity in STED.^[^ ^235^, ^254^ ^]^

New technologies such as the ExoView R200 instruments help address these challenges. ExoView R200 (NanoView Biosciences) is an EV‐specific instrument that characterizes vesicle count, size, and surface markers without the need for sample purification.^[^ ^255^ ^]^ The instrument uses antibodies such as anti‐tetraspanins on the chip to capture exosomes, then uses fluorescent antibodies against multiple targets of interest to measure surface protein expression levels. The ExoView R200 also uses interferometric imaging to measure the sizes of exosomes as small as 50 nm. ExoView R200 offers a more convenient and streamlined solution for exosome characterization than most other methods. However, instrument throughput is limited to processing 16 samples at one time, and the instrument cannot separate different exosome populations for downstream analysis.

Microfluidics techniques have been used in exosome sample preparation and isolation (Section 4.2.6) and for characterizing the physical, biological, and molecular properties of exosomes.^[^ ^256^ ^]^ Instead of retooling existing technologies, the approach streamlines characterization by designing new tools specifically for exosome characterization. These microfluidic platforms can achieve precise single particle‐level analysis by adopting working principles of existing exosome isolation and characterization approaches such as acoustic nanofiltration, deterministic lateral displacement, viscoelastic flow sorting, plasmonic sensing and electrochemical sensing.^[^ ^238^, ^257^ ^]^ This allows researchers to integrate a selection of cutting‐edge technologies on a single device to target specific exosome characterization needs. For example, the plasmonic sensors introduced in Section 5.2.1 can be integrated into microfluidic systems to enables real‐time, label‐free characterization of exosomal membrane proteins at high detection sensitivity.^[^ ^177^, ^258^ ^]^ Another example is the electrochemical sensors that use binding targets such as aptamers to capture exosomes and generate electrical signals for characterization. Existing electrochemical sensor approaches have demonstrated highly sensitive in exosome characterization, with the potential to be scaled‐up for high‐throughput analysis.^[^ ^177^, ^258^ ^]^ By integrating these state‐of‐the‐art technologies into a microfluidic system, high throughput, in situ isolation and analysis can be achieved without the need to switch across instruments while evaluating multiple exosome characteristics.^[^ ^177^, ^258^ ^]^ With the integration, and smaller processing volume and reagent use, user sample handling and transfer can be minimized. As a result, the application of microfluidic systems in exosome studies can potentially provide researchers more streamlined and specialized analytical systems with lower cost and higher accuracy.

Machine learning algorithms offer a way to address challenges in exosome characterization concerning heterogeneity in disease expression across individuals. Machine learning‐based methods have been used to identify and categorize exosomal biomarkers and morphological features and to aid in spectroscopic analysis,^[^ ^243^, ^259^ ^]^ and to assist in applying exosomes as a cancer diagnostic tool, a major area of focus. With more precise isolation and characterization of exosome, researchers will be able to better understand exosome functions and properties, and can apply these findings to develop better exosome‐based diagnostic and therapeutic tools.

### 6.6 Exosome Characterization Methods for Biomedical Applications

Exosome characterization for biomedical applications largely follows the workflow in Scheme 3. Quantitative characterization is focused on total exosome count, total protein, and exosome‐specific markers (proteins and nucleic acids). Total exosome count is assessed by nanoparticle tracking analysis,^[^ ^260^ ^]^ particle morphology and size with transmission electron microscopy^[^ ^261^ ^]^ and DLS,^[^ ^262^ ^]^ respectively. Total protein from isolated exosomes is quantified using a BCA assay.^[^ ^261c^ ^]^ The exosome count indicates the yield, and the ratio of total exosomes to proteins defines the purity of the sample. Exosome protein counts are measured by using Western blot, ELISA, mass spectrometry, and flow cytometry,^[^ ^1^, ^67^, ^260^, ^261^, ^263^ ^]^ and exosomal nucleic acids are quantified with microarrays, NGS, and qPCR.^[^ ^5a^ ^]^ This section will describe exosome characterization in the context of cardiovascular disease, neurodegenerative disease, and HIV/AIDS.

#### 6.6.1 Cardiovascular Diseases

Exosomes isolated from patients with CVD, from the serum of hypertensive rats,^[^ ^1c^ ^]^ or from cultured cardiomyocytes^[^ ^67^, ^263^ ^]^ can be characterized to study and diagnose cardiovascular diseases. CVD‐specific exosomal proteins such as Hsp60 and GAPDH, which are involved in cardiomyocyte apoptosis,^[^ ^263^ ^]^ are characterized by Western blot and mass spectroscopy. CVD‐specific exosomal miRNA, which is released earlier than CVD protein markers and can potentially be used to diagnose early‐stage disease,^[^ ^264^, ^265^ ^]^ can be quantified by RT‐qPCR, and includes the anti‐inflammatory miR‐17 and antiangiogenic miR‐320.^[^ ^1^, ^67^ ^]^

Microfluidic devices provide novel alternate approaches for characterizing CVD‐specific exosomal miRNA markers.^[^ ^1a^ ^]^ Cheng et al. developed a microfluidic chip for detecting miR‐21 and miR‐126,^[^ ^1^ ^]^ which are proangiogenic and cardioprotective, from serum samples. The device integrates exosome isolation and miRNA extraction with detection using antibody‐coated magnetic beads and field effect transistors (FETs). CVD‐related miRNAs are present in blood at very low (e.g., fm) concentrations, and the FET sensors achieve a high sensitivity for detecting immobilized RNA markers by measuring current gain.^[^ ^1^, ^265^ ^]^ The microfluidic device detected miR‐21 and miR‐126 with a limit of detection in the femtomolar range and a total workflow of only 5 h. Although microfluidic devices designed for CVD‐specific exosomal miRNA characterization such as this are not yet well‐established, these studies demonstrate the potential for applying microfluidic devices to exosomal studies and CVD diagnosis.

#### 6.6.2 Neurodegenerative Diseases

Neurodegenerative diseases are prone to misdiagnosis in their early stages due to a lack of knowledge of the pathogenesis mechanisms and a lack of suitable markers for early diagnosis.^[^ ^261a^ ^]^ Exosome detection in Parkinson's and Alzheimer's diseases is potentially useful for improving the early diagnosis and tracking of these diseases.^[^ ^266^ ^]^ Indeed, analysis of exosomes from CSF supports the use of exosomes for studying neurodegenerative disease progression.^[^ ^190^, ^267^ ^]^

Characterization of exosomes isolated from neurodegenerative disease samples for disease diagnosis and basic research includes analysis of the protein markers *α*‐syn^[^ ^261c^ ^]^ and tau^[^ ^262^ ^]^ by mass spectroscopy and immunoassay, and RT‐PCR analysis of dysregulated exosomal RNAs such as miR‐132, which provides neuroprotection for tauopathies (disorders characterized by deposition of abnormal tau protein in the brain)^[^ ^268^ ^]^ and is downregulated in plasma‐derived neurogenic exosomes from AD patients.^[^ ^269^ ^]^

Two challenges in characterizing exosomes in the context of neurodegenerative diseases are the limited sample volume of CSF and the abundance of nanoparticles in CSF that are similar to exosomes in size and density,^[^ ^270^ ^]^ contaminate isolated exosome samples, and cannot be distinguished by NTA.^[^ ^271^ ^]^ Novel methods have been developed to address these challenges.^[^ ^233^, ^272^ ^]^ Stuendl et al. developed a customized ELISA that uses only 0.5 mL of patient CSF to quantify exosomal *α*‐syn with high sensitivity; this assay is based on electrochemiluminescence detection.^[^ ^273^ ^]^ Vandendriessche et al. used an ExoView R100 platform to differentiate exosomes from the abundant nonvesicular particles in CSF in an Alzheimer's mouse model.^[^ ^274^ ^]^ They found that only a small fraction of CSF particles identified by NTA were CD9^+^/CD81^+^ extracullular vesicles. Choroid plexus‐specific CSF EVs were identified by using anti‐transthyretin antibody. ExoView combines immunodetection with imaging techniques and requires only a small sample volume,^[^ ^271^ ^]^ and is a promising detection method for characterizing CSF‐derived exosomes.

#### 6.6.3 HIV/AIDS

Exosomes have played a key role in identifying biomarkers and drug delivery pathways in HIV/AIDS diagnosis and therapeutic research.^[^ ^5^ ^]^ Exosomes have been isolated from HIV/AIDS samples including semen,^[^ ^5a^ ^]^ blood,^[^ ^260^ ^]^ and HIV‐transfected HEK293 cells.^[^ ^275^ ^]^ HIV/AIDS‐specific exosomal protein markers include CD63, CD81, CD9, Nef protein, and acetylcholine esterase, which have been characterized by STED microscopy, SPRM, flow cytometry, Western blot, ELISA, and mass spectrometry.^[^ ^5a^ ^]^ HIV/AIDS‐specific exosomal nucleic acid markers have been characterized by using microarrays, NGS, and qPCR.^[^ ^260^ ^]^ Structural and functional characterization of HIV/AIDS sample‐derived exosomes has been performed by using AFM, SAXS, and SEM.

A promising new platform for exosome characterization is the ExoView R100 platform. Although there are few publications describing the characterization of HIV/AIDS exosome samples using this platform, it has been used to characterize EVs in the context of other viral infectious diseases such as herpes simplex^[^ ^276^ ^]^ and COVID‐19.^[^ ^277^ ^]^ This platform was used to characterize EVs from herpes simplex 1 virus (HSV‐1)‐infected patients, by measuring levels of the tetraspanins CD63, CD81, and CD9.^[^ ^276^ ^]^ The platform was also used to characterize EV CD9, CD63, and CD81 to support the development of therapeutic approaches for blocking SARS‐CoV‐2 cell binding.^[^ ^277^ ^]^ The use of the ExoView R100 platform and other novel integrated miniature devices, such as microfluidic systems with plasmonic and electrochemical sensors to analyze exosomes for disease detection and therapeutics research,^[^ ^276^, ^277^, ^278^ ^]^ can minimize the transfer of samples between instruments when multiple exosome characteristics are studied.

## 7 Summary and Perspective

Exosome research and development efforts have been quite successful at detailing many aspects of exosome structure and functions, their biology, and their contributions to and influences on many disease states. This explosion of information has provided much enthusiasm for utilizing exosomes in disease diagnostics and as therapeutic agents. However, as discussed in this review, exosome research and development efforts have many significant hurdles before they can be as routinely and effectively utilized as other biologic agents in the medical sciences. In particular, suitable exosome production, isolation, downstream purification, and analytical analysis efforts will be required to properly and effectively utilize them in diagnostic applications and as safe and effective therapeutic agents. Overcoming the various manufacturing and diagnostic hurdles is not only a technical matter, but as we discussed, one of guidance and guidelines. As we strive to effectively navigate the scientific and regulatory spaces alike, collaborative efforts from many different fields of basic science, medical sciences, technology development, and the agencies and organizations that financially support and govern these efforts will play significant roles in the speed and success of bringing exosomes forward to the quality and effectiveness of other biologics.

## Conflict of Interest

The authors declare no conflict of interest.
